# Public preferences regarding the priority setting criteria of health interventions for budget allocation: results of a survey of Iranian adults

**DOI:** 10.1186/s12889-022-14404-1

**Published:** 2022-11-08

**Authors:** Ali Darvishi, Rajabali Daroudi, Mehdi Yaseri, Ali Akbari Sari

**Affiliations:** 1 Department of Health Management and Economics, School of Public Health, Tehran University of Medical Sciences, Poursina Ave, Tehran, 1417613151 Iran; 2 Department of Epidemiology and Biostatistics, School of Public Health, Tehran University of Medical Sciences, Poursina Ave, Tehran, 1417613151 Iran; 3 Department of Health Management and Economics, School of Public Health, Tehran University of Medical Sciences, Poursina Ave, Tehran, 1411713137 Iran

**Keywords:** Public preferences, Resource allocation, Priority setting criteria, Health interventions, Iran

## Abstract

**Objectives:**

Priority setting in health directly impacts the general public as payers and final consumers, so the public preferences must be considered. The present study aimed to provide public preferences about health intervention allocation criteria for the optimal allocation of public health budget in Iran.

**Methods:**

A choice-based survey method was used to assess the general public’s preferences regarding 8 critical criteria with a societal aspect. One thousand sixty-four adult citizens of Tehran, Iran, participated in the study. Participants were asked to allocate a hypothetical budget between the two groups with differences in allocation criteria. Public preferences were inferred from absolute majority responses i.e., more than 50% of participants’ allocation for a criterion. The Logistic Regression Model was used to investigate the factors affecting the preferences regarding the importance of allocation criteria.

**Results:**

Based on expressed participants’ preferences, criteria of disease severity, age, daily care needs, Number of alternative interventions, individual’s economic status, and diseases with absence from work were important. Thus, 77, 69, 61, 57, 54, and 51% of participants preferred to allocate the hypothetical budget to the treatment of patients with poor economic status, treatment of patients with diseases leading to absence from work, treating patients with severe diseases, treatment of diseases in need of daily care and treatment of children’s diseases, respectively. Findings from the factors affecting participants’ preferences regarding allocation criteria also showed that people with different characteristics had different preferences.

**Conclusions:**

Iranian general public pays special attention to the criteria of equitable allocation, including patients’ economic status, criteria with societal aspects such as absenteeism from work and the need for daily care, as well as criteria with medical aspects such as disease severity and access to alternative interventions which may sometimes be less considered in decision making.

**Supplementary Information:**

The online version contains supplementary material available at 10.1186/s12889-022-14404-1.

## Introduction

Making decisions in health care is usually very difficult and is always done with great complexity and the trade-off between different goals. This issue is critical in prioritizing health interventions and technologies for public budget allocation and has always been one of the main challenges for decision-makers in this field. Usually, in many countries, including Iran, such decisions and prioritization are based on explicit criteria, including effectiveness, cost, and budget impact of interventions. Usually, other relevant and important allocation criteria are neglected or not explicitly included in the decisions [1].

The advantage of health technology assessment (HTA) and economic evaluation studies is that these offer an output called incremental cost-effectiveness ratio (ICER) that makes different prioritization interventions comparable based on specific thresholds. However, the limitation and concern in this regard are that the ICER criterion considers only criteria of cost and effectiveness and does not consider other important criteria related to interventions in the prioritization. It makes some valuable alternative interventions out of the priority of resource allocation [2].

Although the importance of cost-effectiveness in decision-making is very high, designing and applying decision models based on different characteristics of interventions can lead to better efficiency and fairness in policymaking. An alternative approach to removing these limitations in prioritizing interventions is to use multi-criteria decision models (MCDAs). Several criteria are used in the MCDA method that has been considered in recent decades instead of using one measure of optimality. Developing a multi-criteria approach to prioritization in developing countries has been considered an important issue in health system research [3]. MCDA is a scientific method that considers and evaluates multiple goals, aspects, and criteria along with the cost and effectiveness of decision interventions [4]. For example, other aspects can be the severity of the disease, the criteria of justice and inequality, patients’ economic status, access to appropriate alternatives, the population size, lost productivity of disease, and so on. It should be noted that about HTA and economic evaluation, MCDA has not yet been widely used. However, reputable scientific institutes have presented MCDA models using different criteria and cost-effectiveness [5, 6].

In traditional decision-making approaches, where judgments and decisions are based solely on ICER and not considering the other important criteria associated with interventions, information is not provided on the community’s view of distributive justice. Given that the people of society as the main owner of resources are the main payer and end-user of resources, it can be said that the general public’s point of view and social values, along with the point of view of other stakeholders in this regard is very important and should be considered in decision making. For example, we can mention some interventions such as medicines for rare diseases or some interventions related to cancers, whose ICER levels are usually higher than the cost-effectiveness threshold of countries. However, we see that sometimes the countries’ healthcare system are placed in the Benefit Package, which can be said to have happened by considering some social values related to these interventions.

In recent years, moral, economic, political, and legal arguments in support of paying attention to public preferences in decision-making in health interventions and technologies and setting priorities have been growing [7–10]. It has necessitated the involvement of public opinion in health care decision-making and prioritization decisions among researchers and policymakers [11, 12]. Recognizing and integrating public preferences and participation in the allocation of resources for health interventions is an important step in ensuring the legitimacy, usefulness, and fairness of decisions, and may reduce disputes between people and payers over public budget allocations [13–15].

Given that the decision to finance new interventions and resource allocation to health intervention has a direct impact on the general public in terms of cost and access restrictions, it is essential that the preferences of all stakeholders in the various aspects of interventions along with the needs of patients to be assessed and considered. Information and knowledge about public preferences and valuations help policy, and decision-makers better understand society’s important issues. Therefore, the present study aimed to provide a clear picture of public preferences regarding allocation criteria and provide practical evidence for the optimal allocation of public health resources in Iran.

## Materials and methods

### Study design and participants

The present descriptive-analytical and cross-sectional study were conducted using an extensive survey in 2021 in Tehran, the capital of Iran. In this study, we sought to determine the general public viewpoints about the importance of various criteria for allocating public health budgets. At first, eight important criteria with a social aspect were selected from a set of criteria. Then by designing a survey, people’s opinions and preferences about the importance of these criteria were extracted in different scenarios.

This study’s target population includes Tehran citizens over 18 years old at the time of the survey. Considering that Tehran is the most populous city in Iran, and on the other hand, it includes almost all Iranian cultures and ethnicities, it seems to be the best representative of the whole country.

### Criteria selection and survey design

The most important allocation criteria in their prioritization for budget allocation were extracted and selected. For this purpose, first, by literature review, a set of criteria (31 criteria) introduced in various studies and texts was extracted and summarized. After listing the criteria, classifying them, and removing duplicates and overlaps, 15 were selected as the main criteria. Finally, the eight final criteria for the present study were selected among the 15 main criteria based on group discussion by the study team. In this regard, it was tried to consider the best and the most important criteria according to the context and objectives of the study, which has the most social aspects and distributive justice. Also, due to the survey technique limitations and the time-consuming process, it was not possible to use all the criteria in the study.

The selected criteria included the following:

Disease severity, Age (Children or Adults), Daily care needs, Number of alternative interventions, Individual’s economic status, Population size (common or rare disease), Disease with absence from a job, and Lifestyle-related diseases. Detailed definitions and descriptions of the criteria can be seen in Table 1.

**Table 1:** Criteria descriptions

	Criteria	Description
1	**Disease severity**	The fatality and disability level of the disease
2	**Age (Children or Adults)**	The age range of patients using the intervention (children or adults)
3	**Daily care Needs**	Does the patient need daily care from family members or nurses?
4	**Access to Alternative Interventions**	The number of medicines or alternative interventions available to treat a given disease
5	**Individual’s Economic status**	The disease is more prevalent among the poorer groups of society or groups with good economic status
6	**Population Size (Common or Rare Diseases)**	The number of patients who need specific intervention to treat the disease
7	**Diseases with absence from work**	The impact of illness on absenteeism of working patients
8	**Lifestyle-related diseases**	To what extent people’s unhealthy lifestyle caused the disease

A choice-based survey was used to assess the general public’s preferences regarding allocation criteria. It should be noted that to assess the preferences in health, different methods are used, each of which has its characteristics and limitations. The technique used in the present study is based on two studies conducted in UK and Australia in 2012 and 2018 [16, 17]. Due to the number of criteria and on the other hand, since the research community includes the general public, the method and questionnaire used allowed researchers to ask questions and scenarios in the simplest possible way, and the data collection has a higher quality. Another point is that in this questionnaire, three questions are designed for each criterion, which in fact, will be two of the three questions related to the trade-off of the criteria with the cost and effectiveness.

The questionnaire was designed in such a way that as a scenario for each criterion and through this, people’s preferences regarding the importance of the criteria and the trade-off between cost and effectiveness were measured. The general structure of the questionnaire is as follows:

The first part of the questionnaire was related to basic information about the nature of the research and its objectives, as well as how to answer the research questions.

The second part will be the main scenarios and questions of the study regarding the individuals’ preferences regarding the importance of resource allocation criteria. Three scenarios (questions) are designed for each criterion. (To better explain the scenarios and questions of the questionnaire, a criterion called “disease severity” is given below as an example).

A scenario based on the initial assumptions about the criterion assuming that all the features related to the interventions are equal and fixed (all else being equal). A scenario is adjusted based on the fact that the effectiveness of the hypothetical interventions is different. Finally, a scenario is considered based on the cost changes of the interventions compared to the first scenario (the cost of one intervention is twice the cost of the other intervention, but the effectiveness is the same). These two scenarios are designed to measure changes (trade-offs) in societal preferences due to initial assumptions about effectiveness and cost changes.

Based on the explanations, three scenarios are designed for each criterion and are divided into two cohorts so that in both groups, the first scenario (all else being equal) is fixed; in cohort 1 the second scenario is placed (effectiveness trade-off), and in cohort 2 the third scenario is placed (cost trade-off). Therefore, two editions of the questionnaire will be the same in both groups of the first scenario. The division of scenarios into two cohorts is because the required sample size is reduced, and on the other hand, the respondents are tired due to the high volume of questions. Table 2 provides a complete description of the criteria and questions in each scenario.

**Table 2:** Allocation criteria explored, including Cost and Effectiveness Trade-off scenarios

Criteria	Baseline Scenario: All else being equal (equal treatment costs and effectiveness)	Effectiveness Trade-off Scenario	Cost Trade-off Scenario
Disease severity	Should more budget go to patients with severe disease (Group 1) than those with mild disease (Group 2)?	Smaller treatment effectiveness for severe disease (Group 1) compared with mild disease (Group 2)	Higher costs of treatment for severe disease (Group 1) compared with mild disease (Group 2)
Age (Children or Adults)	Should more budget go to children (Group 1) than adult patients (Group 2)?	Smaller treatment effectiveness for children’s diseases (Group 1) compared with adult diseases (Group 2)	Higher costs of treatment for children’s diseases (Group 1) compared with adult diseases (Group 2)
Daily care Needs	Should more budget go to patients dependent on carers for daily tasks (Group 1) compared to those who are not dependent (Group 2)?	Smaller treatment effectiveness for patients who are dependent on carers for daily tasks (Group 1) compared to those who are not dependent (Group 2)	Higher costs of treatment for patients who are dependent on carers for daily tasks (Group 1) compared to those who are not dependent (Group 2)
Access to Alternative Interventions	Should more budget go to patients for whom there is no alternative available (Group 1) compared to those for whom there are several alternatives (Group 2)?	Smaller treatment effectiveness for patients for whom there is no alternative available (Group 1) compared to those for whom there are several alternatives (Group 2)	Higher costs of treatment for patients for whom there is no alternative available (Group 1) compared to those for whom there are several alternatives (Group 2)
Individual’s Economic status	Should more budget go to economically disadvantaged patients (Group 1) compared to those who are economically well off (Group 2)?	Smaller treatment effectiveness for economically disadvantaged patients (Group 1) compared to those who are economically well off (Group 2)	Higher costs of treatment for economically disadvantaged patients (Group 1) compared to those who are economically well off (Group 2)
Population Size (Common or Rare Diseases)	Should more budget go to patients with rare disease (Group 1) compared to those with common diseases (Group 2)?	Smaller treatment effectiveness for patients with rare disease (Group 1) compared to those with common diseases (Group 2)	Higher costs of treatment for patients with rare disease (Group 1) compared to those with common diseases (Group 2)
Diseases with Absence from work	Should more budget go to patients whose diseases affect their ability to work (Group 1) compared to those who are able to continue working (Group 2)?	Smaller treatment effectiveness for patients whose diseases affect their ability to work (Group 1) compared to those who are able to continue working (Group 2)	Higher costs of treatment for patients whose diseases affect their ability to work (Group 1) compared to those who are able to continue working (Group 2)
Lifestyle-related diseases	Should more budget go to patients with an unrelated lifestyle disease (Group 1) compared to those with a related lifestyle disease (Group 2)?	Smaller treatment effectiveness for patients with an unrelated lifestyle disease (Group 1) compared to those with a related lifestyle disease (Group 2)	Higher costs of treatment for patients with an unrelated lifestyle disease (Group 1) compared to those with a related lifestyle disease (Group 2)

The third part was questions related to characteristics, demographic variables, socioeconomic status and individuals’ health status.

#### Pilot study

As mentioned, the questionnaire design was based on two studies conducted in the past [16, 17] and also based on the summary of the research team’s opinion and research context. After designing the questionnaire, to examine the weaknesses and on the other hand, how the scenarios and questions are understandable to the respondents, and also the average time to answer all the questions in each edition, a pilot study was conducted in terms of 68 samples from the participants and the weaknesses of the questionnaire were eliminated.

(Questionnaire of both cohorts is available in the supplementary material section).

### Data collection method

A face-to-face interview was used to collect data, and two interviewers were trained during the sessions. The data collection was performed in Tehran’s social security insurance centers and government counter offices.

In Iran, more than 90% of people are covered by basic health insurance. This population is mainly covered by social security insurance and Iran health insurance, where the covering share of each is almost equal [18]. To follow up the relevant administrative affairs, social security insurance and government counter offices for Iran health insurance have been considered, and the insured persons refer to these centers to do their tasks, including renewing and changing the insurance booklets. On the other hand, other administrative affairs are done in government counter offices, and some people go to these centers for other tasks. These offices and centers are distributed in different geographical parts of the city. According to these explanations, these centers seemed to be the best place to collect study data according to the goals.

To collect data, from May 2021 to August 2021, interviewers were present alternately in these centers in different geographical locations after obtaining informed consent to collect data for those who could complete the questionnaire personally; the questionnaire was provided to them, and information was collected from other people in the form of interviews. We had no incentive offer for participants to participate in the study.

### Statistical analysis

The data were analyzed so that in the first scenario related to each criterion (all else being equal scenario), the percentage of responses to each of the three groups of patients was determined. For example, in terms of disease severity criteria, respondents could allocate the budget more to patients with high disease severity (Group 1) or patients with mild disease severity (Group 2) or divide the budget equally between the two groups.

In the second and third scenarios of each criterion (effectiveness and cost trade-off questions), we sought to examine the changes in community preferences and their significance by distinguishing between the effectiveness and cost of hypothetical interventions. This section aims to determine the community’s preferences regarding the change of two main criteria of intervention effectiveness and cost, taking into account other related criteria. To determine these changes in preferences over the preferences expressed in the first scenario of each cohort as a basis, the odds ratio (OR) was calculated. Mc-Nemar’s non-parametric test was used to determine the significance of these changes in each cohort for each criterion. The Random intercept Logistic Regression Model was used to investigate the respondent characteristic affecting the preferences regarding the importance of allocation criteria. Stata 14 software was used for calculations and statistical analysis, estimating odds ratio and significant Mc-Nemar tests, and logistic regression models.

## Results

A total of 1064 eligible adults completed the questionnaires. Of these, 962 samples were entered into the final phase and calculations by removing several questionnaires that the answers were not of good quality or were not fully completed. Of these, 491 and 471 were in cohorts 1 and 2, respectively. The average age of the people was 34 years old, most of whom were 25 to 34 years old. 55% were women, and 69% had a university degree or higher. Other information and demographic characteristics of the study participants can be seen in Table 3. Also, all the information is given separately in cohorts 1 and 2, and there is no significant difference between the two groups.

**Table 3:** Characteristics of study respondents

Characteristics	Total Population		Cohort1		Cohort2		Iran (%)*
	Freq.	Percent	Freq.	Percent	Freq.	Percent	
Sample size	962		491		471		
	**Mean**	**SD**	**Mean**	**SD**	**Mean**	**SD**	
Mean Age	34.01	0.29	34.51	0.43	33.49	0.39	
Age Categorized							
18 to 24	119	12.37	60	12.22	59	12.53	16.52
25 to 34	477	49.58	237	48.27	240	50.96	30.71
35 to 44	234	24.32	116	23.63	118	25.05	20.57
44 to 54	98	10.19	57	11.61	41	8.7	14.83
> 55	34	3.53	21	4.28	13	2.76	17.35
Sex							
Male	424	44.07	201	40.94	223	47.35	50.53
Female	538	55.93	290	59.06	248	52.65	49.47
Marital Status							
Single	395	41.06	206	41.96	189	40.13	
Married	546	56.76	274	55.8	272	57.75	
Separated	21	2.18	11	2.24	10	2.12	
Race							
Turk	175	18.19	74	15.07	101	21.44	
Lor	136	14.14	94	19.14	42	8.92	
Kurd	79	8.21	44	8.96	35	7.43	
Mazani/Gilak	137	14.24	55	11.2	82	17.41	
Other	435	45.22	224	45.62	211	44.8	
Religion							
Shia	833	86.59	432	87.98	401	85.14	
Sunni	50	5.2	24	4.89	26	5.52	
Zoroastrianism	17	1.77	4	0.81	13	2.76	
Others	62	6.44	31	6.31	31	6.58	
Education Status							
Primary/ Some High school	18	1.87	8	1.63	10	2.12	
Diploma	182	18.92	92	18.74	90	19.11	
Associate Diploma	94	9.77	51	10.39	43	9.13	
Bachelor	388	40.33	201	40.94	187	39.7	
MSc, Ph.D., MD	280	29.11	139	28.31	141	29.94	
Employment Status							
Working	576	59.88	289	58.86	287	60.93	
Unemployed	54	5.61	35	7.13	19	4.03	
Retired with Income	24	2.49	17	3.46	7	1.49	
Housekeeper	179	18.61	83	16.9	96	20.38	
Student/Soldier	129	13.41	67	13.65	62	13.16	
Insurance							
Not Insured	101	10.5	51	10.39	50	10.62	
Social Security	536	55.72	278	56.62	258	54.78	
National Health Insurance	226	23.49	110	22.4	116	24.63	
Army Forces Insurance	56	5.82	18	3.67	38	8.07	
Others	43	4.47	34	6.92	9	1.91	
Health Status							
very good	482	50.1	254	51.73	228	48.41	
Good	341	35.45	175	35.64	166	35.24	
Average	106	11.02	53	10.79	53	11.25	
Poor	33	3.43	9	1.83	24	5.1	
Having any Rare and Severe Disease (History in Family)	767	79.73	376	76.58	391	83.01	
Household Monthly Expenditure (IR Rial)							
< 20,000,000	55	5.72	28	5.7	27	5.73	
20,000,000-40,000,000	234	24.32	113	23.01	121	25.69	
40,000,000-60,000,000	258	26.82	124	25.25	134	28.45	
60,000,000-80,000,000	261	27.13	123	25.05	138	29.3	
> 80,000,000	154	16.01	103	20.98	51	10.83	
Household Monthly Income (IR Rial)							
< 20,000,000	53	5.51	22	4.48	31	6.58	
20,000,000-40,000,000	166	17.26	77	15.68	89	18.9	
40,000,000-60,000,000	217	22.56	131	26.68	86	18.26	
60,000,000-80,000,000	246	25.57	120	24.44	126	26.75	
> 80,000,000	280	29.11	141	28.72	139	29.51	
Evaluation of Household’s Income							
Low	359	37.32	168	34.22	191	40.55	
Medium	586	60.91	313	63.75	273	57.96	
High	17	1.77	10	2.04	7	1.49	

Based on the latest Statistics of the Iranian Statistic Center (ISC). It should be noted that only the country’s up-to-date age and gender statistics were available, and other statistics were either unavailable or not up-to-date

### Participants’ preferences regarding allocation criteria

#### Preferences of all participants: all else being equal scenario

Table 4 shows the preferences of all participants regarding the importance of criteria in all else being equal Scenario for allocating a hypothetical budget between two groups of patients (for example, in the criterion of “disease severity” between patients with severe disease and patients with mild disease) or equal allocation between the two groups.

**Table 4:** Preferences of study participants regarding allocation criteria in “all else being equal” scenario (All Participants)

	Criteria	Proportion (CI)		
		Prioritize Group 1	Equal allocation to both Groups	Prioritize Group 2
1	**Disease severity**	0.616 (0.585-0.646)	0.237(0.211-0.264)	0.146(0.125-0.170)
2	**Age (Children or Adults)**	0.51(0.484-0.548)	0.411 (0.380-0.443)	0.071(0.057-0.089)
3	**Daily care Needs**	0.547(0.516-0.579)	0.304(0.276-0.334)	0.147(0.126-0.171)
4	**Access to Alternative Interventions**	0.572(0.541-0.603)	0.296(0.268-0.325)	0.13(0.111-0.153)
5	**Individual’s Economic status**	0.77 (0.742-0.795)	0.171 (0.148-0.196)	0.058(0.045-0.074)
6	**Population Size (Common or Rare Diseases)**	0.425(0.394-0.456)	0.292(0.264-0.321)	0.282(0.255-0.312)
7	**Diseases with Absence from work**	0.695(0.665-0.723)	0.216(0.191-0.243)	0.088(0.071-0.108)
8	**Lifestyle-related diseases**	0.448(0.416-0.479)	0.404(0.373-0.435)	0.147(0.126-0.171)

It is generally assumed that the criterion is important when more than 50% of the participants allocate the hypothetical budget to group 1 in each criterion scenario (For example, in the criterion of “disease severity” to the patients with severe disease).

**Fig. 1:**
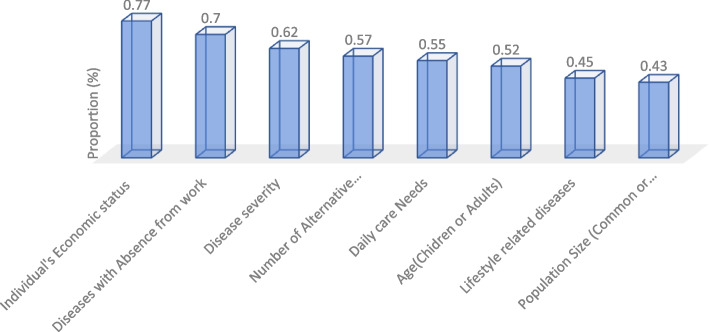
Criteria Ranking based on Participants’ Preferences (all else being equal)

Accordingly, based on participants’ preferences, criteria of disease severity, age, daily care needs, Number of alternative interventions, individual’s economic status, and diseases with Absence from work were important (above 50% of the participants allocated the hypothetical budget to group 1). Thus, participants based on their preferences were allocated a hypothetical budget to the criteria as follows: treatment of severe diseases (61%), treatment of children’s diseases (51%), treatment of diseases in need of daily care (54%), treatment of diseases those have only one therapeutic intervention (57%), treatment of patients with poor economic status (77%), treatment of patients with diseases leading to Absence from work (69%). More details on allocating a budget in all criteria can be seen in Table 4. Also, these results about criteria ranking based on Participants’ Preferences were summarized in Fig. 1. More complete details can be seen in Table S1.

#### Preferences of participants separated by cohorts 1 and 2

Table 5 shows participants’ preferences regarding allocation criteria by cohorts 1 and 2. In this table, in each cohort, preferences are presented based on two scenarios: all else being equal (No trade-off scenario) and the effectiveness or cost of intervention trade-off scenarios (trade-off scenarios).

**Table 5:** Participants’ preferences for allocation criteria (Separating cohorts 1 and 2)

Criteria	Cohort	Choice	Proportion (CI)%		
			Prioritize Group 1	Equal Allocation	Prioritize Group 2
Disease severity	**1**	**No Trade-off**	61.9 (57.5-66.1)	24.6 (21-28.6)	13.4 (10.6-16.7)
		**Effectiveness trade-off**	33.8 (29.7-38.1)	29.9 (26.03-34.15)	36.25 (32.1-40.6)
			OR = 0.314; *p* = 0.001	OR = 1.306; *p* = 0.062	OR = 3.662; *p* = 0.001
	**2**	**No Trade-off**	61.3 (56.8-65.6)	22.7 (19.1-26.7)	15.9 (12.8-19.5)
		**Effectiveness trade-off**	54.9 (50.4-59.4)	28.4 (24.5-32.7)	16.5 (13.4-20.2)
			OR = 0.769; *p* = 0.047	OR = 1.352; *p* = 0.043	OR = 1.047; *p* = 0.791
Age (Children or Adults)	**1**	**No Trade-off**	50.1 (45.6-54.5)	43.7(39.4-48.2)	6.1(4.2-8.6)
		**Effectiveness trade-off**	28.9 (25.06-33.1)	37.8 (33.6-42.2)	33.1 (29.1-37.5)
			OR = 0.405; *p* = 0.001	OR = 0.782; *p* = 0.059	OR = 7.636; *p* = 0.001
	**2**	**No Trade-off**	53.2 (48.7-57.7)	38.4 (34.1-42.9)	8.2 (6.1-11.1)
		**cost trade-off**	61.3 (56.8-65.6)	30.9 (26.9-35.3)	7.6 (5.5-10.4)
			OR = 1.391; *p* = 0.012	OR = 0.719; *p* = 0.016	OR = 0.916; *p* = 0.718
Daily care Needs	**1**	**No Trade-off**	55.6 (51.1-59.9)	28.3 (24.4-32.4)	16.08 (13.08-19.6)
		**Effectiveness trade-off**	35.03 (30.9-39.3)	27.6 (23.9-31.8)	37.2 (33.08-41.6)
			OR = 0.430; *p* = 0.001	OR = 0.97; *p* = 0.831	OR = 3.098; *p* = 0.001
	**2**	**No Trade-off**	53.9 (49.3-58.4)	32.6 (28.5-37.08)	13.3 (10.5-16.7)
		**cost trade-off**	49.4 (44.9-53.9)	33.3 (29.2-37.7)	17.1 (14.04-20.8)
			OR = 0.836; *p* = 0.1709	OR = 1.029; *p* = 0.835	OR = 1.345; *p* = 0.1032
Access to Alternative Interventions	**1**	**No Trade-off**	58.6 (54.2-62.9)	29.3 (25.4-33.5)	12.01 (9.4-15.2)
		**Effectiveness trade-off**	42.1 (37.8-46.5)	28.7 (24.8-32.8)	29.1 (25.2-33.3)
			OR = 0.513; *p* = 0.001	OR = 0.97; *p* = 0.832	OR = 3.008; *p* = 0.001
	**2**	**No Trade-off**	55.8 (51.3-60.2)	29.9 (25.9-34.2)	14.2 (11.3-17.6)
		**cost trade-off**	69.6 (65.3-73.6)	20.1 (16.7-24.05)	10.1 (7.7-13.2)
			OR = 1.814; *p* = 0.001	OR = 0.591; *p* = 0.0005	OR = 3.684; *p* = 0.058
Individual’s Economic status	**1**	**No Trade-off**	78 (74.1-81.4)	17.3 (14.2-20.9)	4.6 (3.1-6.9)
		**Effectiveness trade-off**	57.4 (52.9-61.7)	24.8 (21.2-28.8)	17.7 (14.5-21.3)
			OR = 0.380; *p* = 0.001	OR = 1.579; *p* = 0.003	OR = 4.381; *p* = 0.001
	**2**	**No Trade-off**	76 (71.9-79.6)	16.9 (13.8-20.6)	7 (5.01-9.7)
		**cost trade-off**	77.9 (73.9-81.4)	15.9 (12.8-19.5)	6.1 (4.3-8.7)
			OR = 1.113; *p* = 0.486	OR = 0.925; *p* = 0.66	OR = 0.870; *p* = 0.599
Population Size (Common or Rare Diseases)	**1**	**No Trade-off**	37.4 (33.2-41.8)	30.9 (27.01-35.2)	31.5 (27.5-35.8)
		**Effectiveness trade-off**	31.3 (27.3-35.6)	25.8 (22.1-29.9)	42.7 (38.4-47.2)
			OR = 0.762; *p* = 0.0439	OR = 0.778; *p* = 0.076	OR = 1.62; *p* = 0.0003
	**2**	**No Trade-off**	47.7 (43.2-52.3)	27.3 (23.5-31.6)	24.8 (21.1-28.9)
		**cost trade-off**	53.2 (48.7-57.7)	22.7 (19.1-26.7)	23.9 (20.3-28.07)
			OR = 1.247; *p* = 0.0902	OR = 0.779; *p* = 0.098	OR = 0.955; *p* = 0.761
Diseases with Absence from work	**1**	**No Trade-off**	66.1 (61.8-70.2)	23.6 (20.06-27.6)	10.1 (7.7-13.1)
		**Effectiveness trade-off**	41.3 (37.05-45.7)	27.08 (23.3-31.2)	31.5 (27.5-35.8)
			OR = 0.360; *p* = 0.001	OR = 1.201; *p* = 0.212	OR = 4.068; *p* = 0.001
	**2**	**No Trade-off**	73.03 (68.8-76.8)	19.5 (16.1-23.3)	7.4 (5.3-10.1)
		**cost trade-off**	71.3 (67.07-75.2)	21.4 (17.9-25.3)	7.2 (5.1-9.9)
			OR = 0.918; *p* = 0.560	OR = 1.124; *p* = 0.467	OR = 0.969; *p* = 0.9005
Lifestyle-related diseases	**1**	**No Trade-off**	42.1 (37.8-46.5)	41.1 (36.8-45.5)	16.7 (13.6-20.2)
		**Effectiveness trade-off**	30.7 (26.8-34.9)	32.5 (28.5-36.8)	36.6 (32.4-41.03)
			OR = 0.609; *p* = 0.0002	OR = 0.691; *p* = 0.005	OR = 2.886; *p* = 0.001
	**2**	**No Trade-off**	47.5 (43.06-52.09)	39.7 (35.3-44.2)	12.7 (10.01-16.07)
		**cost trade-off**	52.8 (48.3-57.3)	33.7 (29.6-38.1)	13.3 (10.5-16.7)
			OR = 1.236; *p* = 0.103	OR = 0.774; *p* = 0.058	OR = 1.057; *p* = 0.771

##### Scenario 1: all else being equal (no trade-off scenario)

In this scenario, the results in cohorts 1 and 2 were similar to those in all participants and no significant difference was found. Details of the findings in this regard can be seen in Table 5.

##### Scenario 2: effectiveness trade-off (cohort 1)

In this scenario, participants in cohort 1 (*n* = 491) were asked to reconsider their preferences for scenario 1, assuming that therapeutic intervention was less effective in group 1 and highly effective in second group 2. In this scenario, the same effectiveness of interventions in the two groups of patients is excluded. Changes in participants’ preferences in this scenario compared to the previous scenario as well as its significance based on ORs, are reported in Table 5. OR values indicate changes in the odds of budget allocation in three groups. Odds of budget allocation in group 1 in effectiveness trade-off scenario compared to the all else being an equal scenario in the disease severity, Age, Daily care Needs, Access to Alternative Interventions, Individual’s Economic status, Population Size, Diseases with Absence from work and Lifestyle-related diseases criterion has been significantly^1^ reduced by 69% (OR = 0.31), 59% (OR = 0.405), 57% (OR = 0.43), 48.7% (OR = 0.513), 62% (OR = 0.38), 23.8% (OR = 0.762), 64% (OR = 0.360) and 39.1% (OR = 0.609), respectively.

**Fig. 2:**
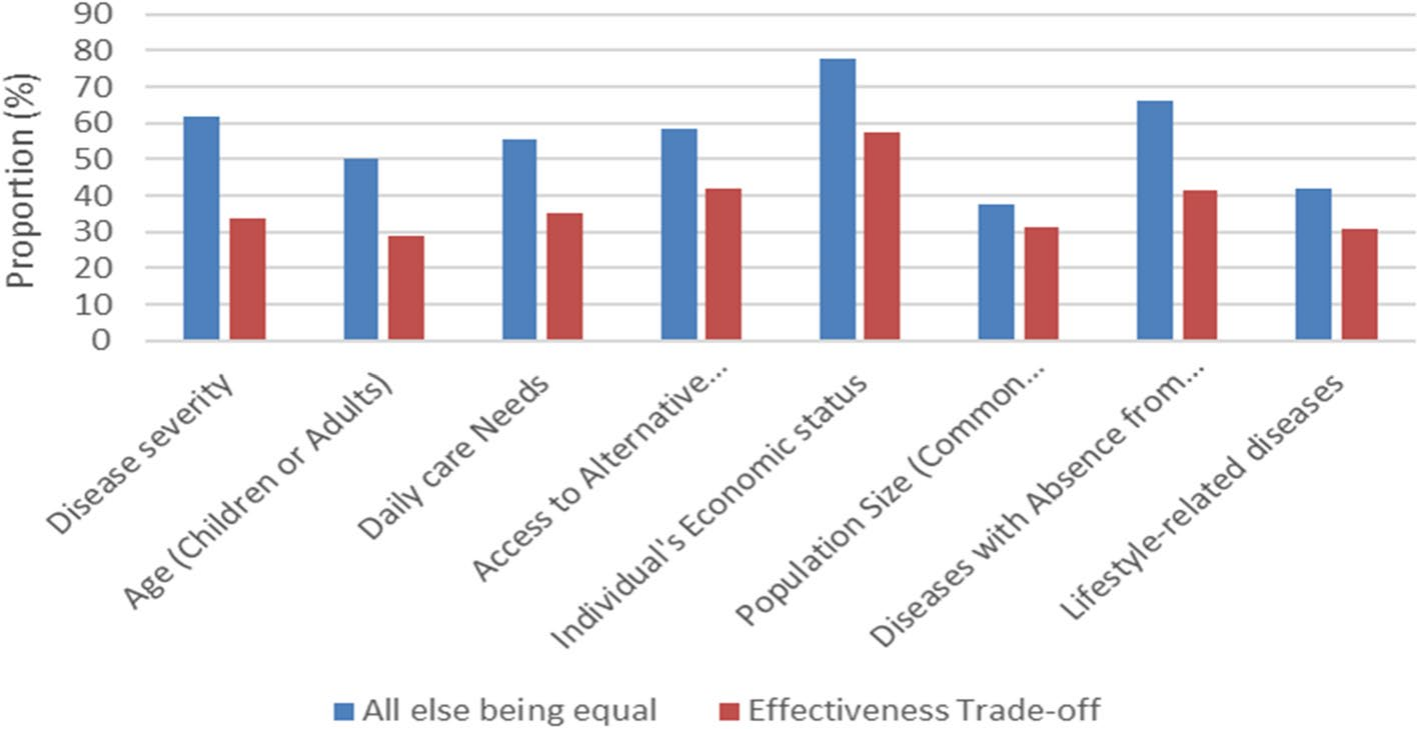
Changing the Participants’ Preferences regarding Allocation Criteria based on “all else being equal” and “effectiveness trade-off” scenarios (Cohort 1)

In this scenario, with different considerations of the effectiveness of interventions, as mentioned participants’ preferences regarding all allocation criteria were significantly shifted. Participants’ preferences shifted from group 1 with low-effectiveness interventions to group 2 with high-effectiveness interventions or equal allocation between two groups. Also, changing the participants’ preferences about allocation criteria based on “all else being equal” and “effectiveness trade-off” scenarios are shown in Fig. 2.

##### Scenario 3: cost trade-off (cohort 2)

In this scenario, participants in cohort 2 (*n* = 471) were asked to reconsider their preferences for Scenario 1, assuming that the cost of treatment intervention in group 1 is twice the cost of treatment intervention the group 2. In this scenario, the assumption that the cost of interventions is the same in the two groups of patients is excluded. Similar to Cohort 1, changes in the participants’ preferences in this scenario compared to scenario 1 and its significance based on ORs are reported in Table 5. In the disease severity criterion, the odds of budget allocation in group 1 in the cost trade-off scenario compared to the all else being equal scenario has been significantly [1] reduced by 23% (OR = 0.769). In return, the odds of budget allocation in group 1 in the Age and Access to Alternative Interventions criterion has been significantly1 increased significantly by 39% (OR = 1.39) and 81% (OR = 1.81), respectively.

**Fig. 3:**
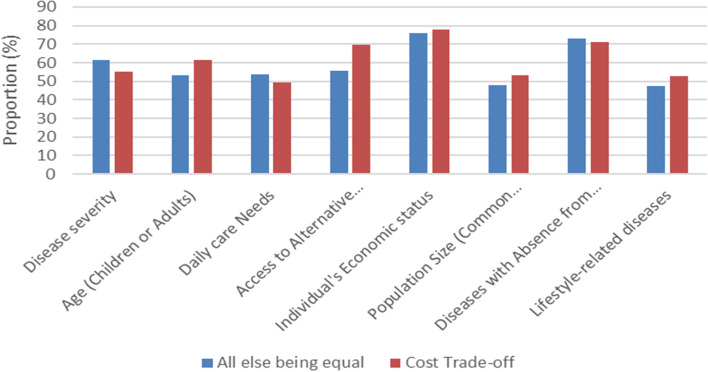
Changing the Cohort 2 Participants’ Preferences regarding Allocation Criteria based on “all else being equal” and “cost trade-off” scenarios (Cohort 2)

In this scenario, with different considerations of the cost of interventions in the patient groups, the changes in participants’ preferences regarding the allocation criteria were different. As mentioned, only the changes in preferences regarding the three criteria were significant. Participants’ preferences regarding “age” and the “access to alternative interventions” shifted to group 1 (interventions at twice the cost). Regarding the “disease severity” criterion, the findings showed significant changes in preferences towards group 2 (lower cost interventions). Similar to scenario 2, changing the participants’ preferences regarding allocation criteria based on “all else being equal” and “cost trade-off” scenarios are shown in Fig. 3.

More complete details can be seen in Table S2.

### Respondents’ characteristics and allocation preferences

Logistic regression was also performed for all eight criteria to identify the factors affecting the participants’ preferences (favoring group 1) based on scenario 1(all else being equal). In this section, we aimed to investigate if there is a difference in results between participants with different characteristics. The findings of this regression analysis are summarized in Table 6. The findings of all eight regressions can be seen in this table. The table structure is such that the columns represent regression and each criterion as the dependent variable, and the rows also represent the explanatory variables (Participants’ characteristics).

**Table 6:** Results of logistic regressions of participants’ characteristics and preferences regarding allocation criteria

Explanatory Variables	Dependent Variables							
	Disease severity, Coefficients(95% CI)	Age (Children or Adults), OR (95% CI)	Daily care Needs, OR (95% CI)	Access to Alternative Interventions, OR (95% CI)	Individual’s Economic status, OR (95% CI)	Population Size (Common or Rare Diseases), OR (95% CI)	Diseases with Absence from work, OR (95% CI)	Lifestyle-related diseases, OR (95% CI)
**Age**								
**18 to 24**	Referent	Referent	Referent	Referent	Referent	Referent	Referent	Referent
**25 to 34**	1.06 (0.62-1.80)	0.62 (0.36-1.04)**	1.48 (0.88-2.5)	1.57 (0.94-2.63)**	1.96 (1.06-3.6)*	0.93 (0.55-1.59)	0.91 (0.52-1.58)	0.78 (0.47-1.3)
**35 to 44**	1.35 (0.73-2.49)	0.32 (0.18-0.59)*	1.28 (0.71-2.32)	1.02 (0.57-1.83)	1.48 (0.75-2.93)	1.04 (0.57-1.88)	0.83 (0.44-1.56)	0.56 (0.31-0.99)*
**45 to 54**	0.41 (0.20-0.83)*	0.25 (0.13-0.51)*	0.89 (0.45-1.79)	0.59 (0.31-1.16)	0.89 (0.41-1.94)	1.86 (0.94-3.69)**	0.46 (0.22-0.94)*	0.69 (0.35-1.34)
**> 55**	1.06 (0.36-3.09)	0.52 (0.18-1.48)	0.61 (0.21-1.79)	1.75 (0.64-4.79)	0.83 (0.27-2.57)	0.50 (0.17-1.46)	1.5 (0.47-4.79)	0.56 (0.2-1.56)
**Sex**								
**Male**	Referent	Referent	Referent	Referent	Referent	Referent	Referent	Referent
**Female**	0.93 (0.68-1.26)	0.71 (0.53-0.96)*	0.78 (0.58-1.06)	0.87 (0.65-1.17)	1.40 (0.98-1.99)**	1.09 (0.81-1.47)	0.81 (0.58-1.12)	0.72 (0.54-0.96)
**Marital Status**								
**Single**	Referent	Referent	Referent	Referent	Referent	Referent	Referent	Referent
**Married**	1.78 (1.26-2.52)*	1.20 (0.86-1.67)	1.69 (1.21-2.37)	1.37 (0.99-1.90)**	1.11 (0.75-1.66)	0.91 (0.65-1.27)	0.77 (0.54-1.1)	0.95 (0.69-1.31)
**Separated**	4.23 (1.28-13.99)*	1.31 (0.49-3.52)	5.82 (1.77-19.11)*	2.83 (0.99-8.11)**	1.01 (0.32-3.19)	2.77 (1.04-7.4)	9.93 (1.24-79.13)	1.84 (0.71-4.79)
**Religion**								
**Shia**	Referent	Referent	Referent	Referent	Referent	Referent	Referent	Referent
**Sunni**	2.75 (1.30-5.78)	0.73 (0.38-1.39)	1.38 (0.72-2.63)	2.23 (1.1-4.52)*	0.83 (0.39-1.74)	2.77 (1.44-5.29)*	0.8 (0.41-1.57)	1.17 (0.62-2.21)
**Zoroastrianism**	1.29 (0.43-3.89)	1.99 (0.64-6.18)	2.23 (0.71-7.01)	2.91 (0.78-10.87)	0.37 (0.12-1.11)**	0.35 (0.11-1.12)**	1	1.31 (0.46-3.69)
**Others**	4.59 (2.14-9.86)	0.58 (0.32-1.05)**	1.67 (0.9-3.12)	1.15 (0.64-2.05)	2.001 (0.87-4.58)	0.83 (0.46-1.5)	1.84 (0.89-3.81)**	1.14 (0.64-2.02)
**Education**								
**Ph.D., MSc, MD**	Referent	Referent	Referent	Referent	Referent	Referent	Referent	Referent
**Bachelor**	0.95(0.66-1.38)	0.93 (0.65-1.33)	1.09 (0.76-1.57)	1.36 (0.95-1.93)**	0.97 (0.63-1.49)	0.58 (0.40-0.83)*	0.46 (0.31-0.7)*	0.84 (0.59-1.20)
**Associate Diploma**	1.02 (0.59-1.77)	0.82 (0.48-1.39)	0.85 (0.49-1.46)	1.66 (0.97-2.84)**	0.58 (0.31-1.05)**	1.28 (0.75-2.18)	0.57 (0.32-1.02)**	0.95 (0.56-1.6)
**Diploma**	0.95 (0.58-1.56)	1.35 (0.84-2.18)	1.55 (0.95-2.52)**	1.56 (0.97-2.51)**	0.92 (0.52-1.63)	1.49 (0.93-2.40)**	0.58 (0.34-0.99)*	0.83 (0.52-1.33)
**Primary/ Some High school**	4.45 (1.06-18.62)*	0.55 (0.17-1.76)	0.72 (0.21-2.41)	1.75 (0.56-5.49)	5.88 (0.67-51.25)	1.65 (0.52-5.23)	0.17 (0.05-0.54)*	0.53 (0.16-1.75)
**Employment Status**								
**Working**	Referent	Referent	Referent	Referent	Referent	Referent	Referent	Referent
**Unemployed**	0.55 (0.28-1.05)	0.58 (0.31-1.10)	1.02 (0.54-1.93)	0.68 (0.36-1.27)	1.19 (0.54-2.59)	0.53 (0.27-1.05)**	1.05 (0.53-2.11)	1.24 (0.67-2.33)
**Retired with Income**	0.49 (0.16-1.48)	0.22 (0.06-0.79)**	0.17 (0.04-0.68)*	0.77 (0.27-2.16)	1.11 (0.34-3.62)	0.34 (0.09-1.16)**	0.52 (0.17-1.63)	1.24 (0.44-3.52)
**Housekeeper**	0.79 (0.52-1.23)	0.85 (0.56-1.30)	0.75 (0.49-1.14)	0.78 (0.51-1.19)	0.72 (0.44-1.17)	0.76 (0.5-1.16)	1.44 (0.90-2.31)	0.77 (0.51-1.18)
**Student/Soldier**	0.86 (0.53-1.41)	0.58 (0.36-0.95)	1.7 (1.04-2.77)	0.78 (0.49-1.25)	1.63 (0.89-2.98)	0.96 (0.6-1.55)	0.78 (0.46-1.32)	1.05 (0.66-1.67)
**Insurance**								
**Not Insured**	Referent	Referent	Referent	Referent	Referent	Referent	Referent	Referent
**Social Security**	0.99 (0.58-1.66)	0.91 (0.55-1.52)	1.48 (0.89-2.47)	0.73 (0.44-1.23)	1.06 (0.59-1.91)	0.87 (0.52-1.43)	1.54 (0.89-2.65)	1.27 (0.77-2.10)
**National Health Insurance**	0.93 (0.52-1.63)	0.85 (0.49-1.47)	1.15 (0.66-1.99)	0.94 (0.54-1.64)	1.41 (0.75-2.67)	0.56 (0.32-0.97)	1.64 (0.90-2.99)	1.51 (0.88-2.59)
**Army Forces Insurance**	0.86 (0.41-1.83)	1.09 (0.52-2.30)	0.98 (0.47-2.05)	0.60 (0.29-1.26)	0.52 (0.23-1.15)	0.73 (0.35-1.54)	0.94 (0.43-2.05)	1.14 (0.55-2.36)
**Others**	0.43 (0.19-1.01)	1.02 (0.45-2.32)	0.47 (0.20-1.1)	0.96 (0.43-2.14)	16.32 (2.03-131.32)	1.08 (0.48-2.43)	0.73 (0.31-1.69)	1.27 (0.57-2.8)
**Health status**								
**very good**	Referent	Referent	Referent	Referent	Referent	Referent	Referent	Referent
**good**	1.28 (0.94-1.76)	1.12 (0.83-1.52)	1.77 (1.29-2.42)	1.18 (0.87-1.59)	1.51 (1.05-2.19)	1.02 (0.75-1.38)	1.47 (1.05-2.05)	0.92 (0.69-1.24)
**Average**	0.64 (0.39-1.06)	1.07 (0.66-1.73)	0.83 (0.51-1.34)	1.05 (0.65-1.7)	1.28 (0.72-2.67)	1.26 (0.78-2.04)	1.17 (0.69-1.99)	0.90 (0.56-1.46)
**Poor**	1.44 (0.59-3.54)	4.39 (1.78-10.85)	0.33 (0.13-0.79)	0.71 (0.31-1.60)	0.98 (0.39-2.49)	1.49 (0.65-3.42)	1.51 (0.61-3.75)	2.37 (1.02-5.52)
**Rare and Severe Disease**	1.14 (0.79-1.64)	0.80 (0.56-1.14)	0.93 (0.65-1.34)	0.87 (0.61-1.23)	0.82 (0.55-1.23)	0.94 (0.66-1.35)	1.46 (0.98-2.18)	0.62 (0.44-0.89)
**Household Expenditure**								
**< 20,000,000**	Referent	Referent	Referent	Referent	Referent	Referent	Referent	Referent
**20,000,000-40,000,000**	0.39 (0.16-0.94)	0.19 (0.08-0.44)	0.66 (0.29-1.52)	1.47 (0.67-3.23)	0.74 (0.28-1.95)	0.78 (0.36-1.69)	0.37 (0.14-0.99)	0.69 (0.31-1.51)
**40,000,000-60,000,000**	0.49 (0.19-1.24)	0.25 (0.10-0.61)	0.40 (0.17-0.96)	1.84 (0.81-4.21)	0.94 (0.34-2.6)	0.92 (0.4-2.07)	0.45 (0.16-1.24)	0.53 (0.23-1.22)
**60,000,000-80,000,000**	0.55 (0.22-1.41)	0.18 (0.07-0.45)	0.45 (0.18-1.1)	1.98 (0.85-4.62)	1.23 (0.43-3.47)	0.62 (0.27-1.43)	0.25 (0.09-0.71)	0.44 (0.19-1.03)
**> 80,000,000**	0.65 (0.24-1.71)	0.13 (0.05-0.34)	0.36 (0.14-0.9)	2.14 (0.89-5.19)	0.55 (0.19-1.6)	0.92 (0.38-2.20)	0.19 (0.06-0.55)	0.46 (0.19-1.12)
**Household Income**								
**< 20,000,000**	Referent	Referent	Referent	Referent	Referent	Referent	Referent	Referent
**20,000,000-40,000,000**	1.25 (0.53-2.95)	3.49 (1.46-8.39)	0.76 (0.32-1.79)	0.63 (0.27-1.46)	0.88 (0.31-2.43)	1.76 (0.77-4)	1.52 (0.59-3.92)	1.65 (0.72-3.8)
**40,000,000-60,000,000**	1.03 (0.42-2.55)	6.002 (2.39-15.06)	1.12 (0.46-2.73)	0.76 (0.31-1.84)	0.73 (0.25-2.12)	1.54 (0.65-3.66)	1.05 (0.39-2.85)	3.48 (1.45-8.34)
**60,000,000-80,000,000**	1.57 (0.61-4.03)	4.62 (1.78-11.98)	1.69 (0.67-4.30)	0.69 (0.27-1.72)	0.62 (0.20-1.9)	1.9 (0.77-4.67)	1.45 (0.52-4.08)	3.68 (1.48-9.13)
**> 80,000,000**	0.99 (0.38-2.56)	4.52 (1.71-11.93)	0.96 (0.37-2.45)	0.49 (0.19-1.24)	0.95 (0.31-2.95)	1.67 (0.67-4.18)	1.82 (0.64-5.21)	3.73 (1.48-9.37)
**Evaluation of Household Income**								
**Low**	Referent	Referent	Referent	Referent	Referent	Referent	Referent	Referent
**Medium**	0.99 (0.71-1.38)	0.73 (0.53-1.02)	0.67 (0.48-0.94)	0.92 (0.67-1.27)	0.86 (0.58-1.26)	0.83 (0.6-1.15)	0.77 (0.54-1.11)	0.76 (0.55-1.04)
**High**	6.41 (1.33-30.76)	1.52 (0.49-4.69)	1.08 (0.36-3.23)	1.22 (0.41-3.56)	0.92 (0.27-3.06)	0.27 (0.08-0.92)	0.85 (0.27-2.63)	1.03 (0.35-3)
**Goodness of Fit**								
**LR chi2(40)**	118.76	104.81	128.43	67.84	87.31	89.92	108.13	62
**Prob > chi2**	0	0	0	0.0039	0	0	0	0.0144
**Pseudo R2**	0.0927	0.0787	0.0969	0.0517	0.0842	0.0685	0.0924	0.0469

*P value< 0.05

**P value< 0.1

#### Disease severity

In respondents aged 45 to 54, there was a significantly lower preference for allocating budgets to patients with high disease severity (group 1) than in the referent category. In contrast, there was a greater preference for allocating resources to patients with severe diseases among married and separated individuals and those with a primary education level.

#### Age

Participants in the age category of 25 to 34 years (*P* < 0.1), 35-44 and 45-54 years, people with other religions (*P* < 0.1), retired people (P < 0.1), and also women were significantly less inclined to allocate budgets to children.

#### Daily care needs

Separated individuals and those with a diploma (*P* < 0.1) tended to allocate the budget to patients with daily care needs. Retired individuals were also significantly less likely to allocate the budget to patients with daily care needs.

#### Access to alternative interventions

Sunni participants were more likely to allocate funds to patients with limited access to alternative interventions. Participants with less than postgraduate education (*P* < 0.1) were also more inclined to allocate the budget to patients with limited access to alternative interventions.

#### Individual’s economic status

People aged 25 to 34 years and women (*P* < 0.1) had a higher tendency, and people with Zoroastrian religion had a lower tendency to allocate budget to the poorer patient group.

#### Population size

Participants in the age category of 45 to 54 years (*P* < 0.1), people with Sunni religion, and people with diploma education (*P* < 0.1) were more inclined to allocate resources to patients with rare diseases, and on the other hand people with Zoroastrian religion (*P* < 0.1), people with a bachelor’s degree, and unemployed and retired people (*P* < 0.1) were less inclined to allocate limited budget to groups of patients with rare diseases.

#### Diseases with absence from work

Participants in the 45-54 age category and those with postgraduate education were less likely to allocate budget to patients with diseases with absence from work. Individuals with other religions were more likely to allocate the budget to patients with diseases with absence from work.

#### Lifestyle-related diseases

Participants in the 35-44 age category were less inclined to allocate budget to patients with lifestyle-related diseases than in the referent category.

There were no other significant differences in preferences for any criterion based on respondents’ characteristics. Other details of this section can be seen in Table 6. In this table, the significance of the variables is shown at the level of 5 and 10%.

## Discussion

The purpose of this study was to evaluate the viewpoints of the Iranian general public on how to allocate public health resources to health interventions as payers and final consumers. In this study, we examined the general public’s preferences regarding the various criteria for allocating health budgets.

Considering the preferences of the general public regarding the criteria for allocating resources along with the preferences of other stakeholders can be of great help in increasing social trust and the effectiveness of policies and the long run, aligning the preferences of the two groups of public society and policymakers of a country. This issue has always been considered in the health sector in developed countries and has sometimes had significant results. A study by Whitty et al. in Australia to test the compatibility of the preferences of the public and decision-makers for general drug subsidies showed that the preferences of both groups were almost identical, and both groups considered disease severity as the most important criterion for subsidy allocation [19].

The analysis of this study was performed under different scenarios, including “all else being equal” (No trade-off), effectiveness, and cost trade-off. Also, factors affecting people’s preferences about allocation criteria were examined.

Under the “all else being equal” scenario, the participants of this study identified the individual’s economic status, diseases with absence from work, disease severity, access to alternative interventions, daily care needs, and age as important allocation criteria, respectively. Lifestyle-related diseases and the population size of patients have been evaluated as the least important criteria.

Similar to the present study in the world, two studies have been conducted in the United Kingdom and Australia. In a 2012 study by Linley et al. in the United Kingdom, they aimed to examine societal preferences and perspectives on drug prioritization criteria for funding. The results of this study showed that the respondents significantly supported the criteria of disease severity, appropriate alternatives to drugs, the degree of innovation of drugs, and the broader social benefits, but in contrast to the criteria of end-of-life interventions, child-related interventions, and disadvantaged populations, as well as rare diseases, were not important in terms of society [17]. Another similar study in Australia (2017) conducted by Kim et al. was a large-scale survey to determine the essential factors and criteria for allocating government funding to drugs in Australia. The results showed that the criteria of disease severity, the availability of alternative interventions for the disease, diseases that mostly target low-income families, and diseases that are not related to lifestyle were the most important criteria in terms of society [16]. In a study by Whitty et al. in Australia, both groups of the public and decision-makers considered disease severity as the most important criterion for subsidy allocation [19].

In the present study on the “population size” criterion, participants in the budget allocation allocated less than 50% of the limited budget to people with common diseases based on all else being equal scenarios. A study by Collego et al. (2007) in Australia to assess public perceptions of prioritizing access to high-cost drugs in public hospitals showed that participants took into account factors such as treatment outcomes, quality of life, and current health status in determining who should have access to HCM. Participants considered that resources should be allocated in such a way that a larger population of the community would benefit [20].

With the elimination of the assumptions of all else being equal and changes in the effectiveness and the cost of hypothetical treatment for each criterion, the findings were different, and people’s preferences were particularly sensitive to changes in these variables. Accordingly, the participants’ preferences regarding all allocation criteria were significantly shifted in effectiveness trade-off scenarios. Participants shifted limited budget allocation to high-effectiveness interventions in this case in all criteria. In this scenario, only in the criterion of individual’s economic status, more than 50% of the limited budget was still allocated to group 1. In other criteria, this amount was less than 50%.

In UK and Australia studies, participants’ preferences have shifted towards the populations that gained a `considerable effectiveness’ except for the `end-of-life treatments’ criterion [16, 17].

Assuming that the cost of medical intervention doubled in group 1 of patients compared to group 2, the assumption that the intervention cost was the same in the two groups of patients was excluded. In this case, the changes in participants’ preferences regarding allocation criteria were different, and only the changes in preferences regarding the three criteria of age, access to alternative interventions, and disease severity were significant. Budget allocation in terms of age and access to alternative interventions shifted to interventions with double the cost. In terms of disease severity, the findings show significant changes in preferences for lower-cost interventions. In this case, changes in participants’ preferences regarding budget allocation were not significant in other criteria. The findings of the UK and Australian studies in this scenario were somewhat different from the results of the present study. Participants in the UK and Australia studies expressed a significant shift in preferences towards the populations that were costly treatments for all criteria, except the disease severity criterion [16, 17].

Findings from the factors (individual characteristics) affecting respondents’ preferences regarding allocation criteria also showed that some people with different characteristics had different preferences than other groups and these changes are different in different criteria. People aged 45 to 54 years have a significantly lower preference for allocating budgets to patients with high disease severity. In contrast, there was a greater preference for allocating resources to patients with severe diseases among married and separated individuals and those with a primary education level. Participants aged 25 to 34 years, 35-44, and 45-54 years, people with non-Shiite religions, retired people, and women were less inclined to allocate budgets to children. Separated individuals and those with a diploma education tended to allocate budget to patients with daily care needs. Retired individuals were also less likely to allocate budget to patients with daily care needs. Sunni participants were more likely to allocate budget to patients with no alternative interventions. Participants with less than postgraduate education were also more inclined to allocate budget to patients with limited access to alternative interventions. People aged 25 to 34 years and women tended to allocate budget to the poorer patient group. In return, people with Zoroastrian religion had a lower tendency to allocate budget to this group. Individuals with 45 to 54 years, people with Sunni participants, and people with diploma education had more tendency to allocate resources to patients with rare diseases and on the other hand people with Zoroastrian religion, people with a bachelor’s degree, and unemployed and retired people were less inclined to allocate limited budget to this group of patients. Participants in the 45-54 age category and those with postgraduate education were less likely to allocate budget to patients with diseases who were absent from work. Still, individuals with non-Shiite religions were more likely to allocate budget to these patients. Also, participants aged 35-44 years were less inclined to allocate budget to patients with lifestyle-related diseases. In UK and Australia studies, people with children were more likely to allocate resources to children, cancer, and rare diseases [16, 17]. In Australia study, people with higher incomes were more likely to allocate resources to patients with severe diseases and patients with only one intervention. Also, people who did not have a full-time job were more likely to allocate resources to weaker economic groups, children, and patients with unrelated lifestyle diseases [16].

### Limitations and strengths

Questionnaire simplification and scenario design can be among the limitations of this study. Given that the level of public knowledge about allocation criteria is limited, we tried to extract their preferences with the simplest design, which has its limitations. In this design we assumed only one criterion as a variable in each question. In this study, we did not measure the preferences of individuals in trade-off with each other and only measured all the criteria once with the cost trade-off and once with the effectiveness trade-off.

To avoid wasting time answering the questionnaire questions, we limited the criteria, did not include all the criteria in the study and tried to include the most important criteria with the least overlap and duplication. Also, for this reason, we did not ask trade-off questions from both groups, divided the questionnaire into two categories, and conducted the survey in two cohorts. On the other hand, because the Covid-19 pandemic accompanied the survey of the present study, some questionnaires were completed electronically.

Regarding the sample’s representativeness, the study sample is much younger and there are more female in this study. Although these differences are not substantial but can be considered a limitation of the present study.

The most important strength of this study was the use of a simple design of questions and scenarios to understand the problem accurately by the general public. In addition to its limitations, it helped obtain more accurate findings compared to alternative methods. This issue has been mentioned by researchers in two similar studies in UK and Australia [16, 17]. Another strength of the study was the relatively good statistical population for the research, which helps to some extent in the generalizability of the results. Also, the use of trade-off scenarios in this study in designing questions showed behavioral changes in people’s preferences in trade-off different states, although it seems that these changes in preferences in the trade-off scenarios have been shown better in effectiveness trade-off scenarios.

## Conclusions

The ultimate goal of health systems in the world as the legal responsibility for providing health care services is to try to provide these services efficiently and fairly. Given the limited resources and the need to prioritize the allocation of resources to health interventions, it is important to consider the most important criteria from the general public’s viewpoint. The results of the present study showed that the general public in Iran pays special attention to the criteria of equitable allocation and efficiency. In particular, attention to individuals’ income deciles and patients’ economic status, criteria with societal aspects such as absenteeism from work and the need for daily care, as well as criteria with medical aspects such as disease severity and access to alternative interventions are some of the issues that may sometimes be less considered in decision making. On the other hand, other criteria that power groups may impose should be considered more in prioritizing and allocating resources. Paying attention to the willingness and preferences of the general public can be more effective in public policies, especially in the health sector, because the community’s support in achieving the goals of decisions and policies will be essential. The main and vital challenge in this regard is how to apply these criteria along with other explicit criteria such as safety, efficacy, and criteria such as cost-effectiveness, and budget impacts, which have recently been developed using MCDA models. This issue can be given special attention in Iran in future studies due to the context of the country.

## Supplementary Information

**Additional file 1: Table S1.** Preferences of Study Participants regarding Allocation Criteria in “all else being equal” Scenario (All Participants). **Table S2.** Participants’ Preferences for Allocation Criteria (Separating cohorts 1 and 2).

**Additional file 2: Supplementary Materials.** Questionnaire for Cohort 1. **Supplementary Materials.** Questionnaire for Cohort 2.

## Data Availability

The datasets generated and/or analyzed during the current study are not publicly available but are available from the corresponding author on reasonable request.

## Footnotes

1 *P* value< 0.05
