# Supplementary material for: Public preferences regarding the priority setting criteria of health interventions for budget allocation: results of a survey of Iranian adults
Source: BMC Public Health. 2022 Nov 8;22:2038. doi: 10.1186/s12889-022-14404-1 (PMC9640781; doi:10.1186/s12889-022-14404-1)
Supplement: Supplementary file 1 — Additional file 1: Table S1. Preferences of Study Participants regarding Allocation Criteria in “all else being equal” Scenario (All Participants). Table S2. Participants’ Preferences for Allocation Criteria (Separating cohorts 1 and 2). [file 12889_2022_14404_MOESM1_ESM.docx]

**Supplementary Information:**

**Table S1: Preferences of Study Participants regarding Allocation Criteria in "all else being equal" Scenario (All Participants)**

|  | Criteria |  | Prioritize Group 1 | Equal allocation to both Groups | Prioritize Group 2 |
| --- | --- | --- | --- | --- | --- |
| 1 | **Disease severity** | **N** | 593 | 228 | 141 |
|  |  | **Proportion(CI)** | 0.616 (0.585-0.646) | 0.237(0.211-0.264) | 0.146(0.125-0.170) |
| 2 | **Age (Children or Adults)** | **N** | 497 | 396 | 69 |
|  |  | **Proportion(CI)** | 0.51(0.484-0.548) | 0.411 (0.380-0.443) | 0.071(0.057-0.089) |
| 3 | **Daily care Needs** | **N** | 527 | 293 | 142 |
|  |  | **Proportion(CI)** | 0.547(0.516-0.579) | 0.304(0.276-0.334) | 0.147(0.126-0.171) |
| 4 | **Access to Alternative Interventions** | **N** | 551 | 285 | 126 |
|  |  | **Proportion(CI)** | 0.572(0.541-0.603) | 0.296(0.268-0.325) | 0.13(0.111-0.153) |
| 5 | **Individual's Economic status** | **N** | 741 | 165 | 56 |
|  |  | **Proportion(CI)** | 0.77 (0.742-0.795) | 0.171 (0.148-0.196) | 0.058(0.045-0.074) |
| 6 | **Population Size (Common or Rare Diseases)** | **N** | 409 | 281 | 272 |
|  |  | **Proportion(CI)** | 0.425(0.394-0.456) | 0.292(0.264-0.321) | 0.282(0.255-0.312) |
| 7 | **Diseases with Absence from work** | **N** | 669 | 208 | 85 |
|  |  | **Proportion(CI)** | 0.695(0.665-0.723) | 0.216(0.191-0.243) | 0.088(0.071-0.108) |
| 8 | **Lifestyle-related diseases** | **N** | 431 | 389 | 142 |
|  |  | **Proportion(CI)** | 0.448(0.416-0.479) | 0.404(0.373-0.435) | 0.147(0.126-0.171) |

**Table S2: Participants' Preferences for Allocation Criteria (Separating cohorts 1 and 2)**

| Criteria | Cohort | Choice | Prioritize Group 1 | Equal Allocation | Prioritize Group 2 |
| --- | --- | --- | --- | --- | --- |
| Disease severity | **1** | **No Trade-off** | 305(61.9, 57.5-66.1) | 121(24.6,21-28.6) | 66(13.4, 10.6-16.7) |
|  |  | **Effectiveness trade-off** | 166 (33.8, 29.7-38.1) | 147(29.9, 26.03-34.15) | 179(36.25, 32.1-40.6) |
|  |  |  | OR = 0.314; p=0.001 | OR = 1.306; p=0.062 | OR = 3.662; p=0.001 |
|  | **2** | **No Trade-off** | 289(61.3, 56.8-65.6) | 107(22.7, 19.1-26.7) | 75(15.9, 12.8-19.5) |
|  |  | **Effectiveness trade-off** | 259(54.9,50.4-59.4) | 134(28.4, 24.5-32.7) | 78(16.5, 13.4-20.2) |
|  |  |  | OR = 0.769; p=0.047 | OR = 1.352; p=0.043 | OR = 1.047; p=0.791 |
| Age(Children or Adults) | **1** | **No Trade-off** | 247 (50.1, 45.6-54.5) | 215(43.7, 39.4-48.2) | 30(6.1, 4.2-8.6) |
|  |  | **Effectiveness trade-off** | 142(28.9, 25.06-33.1) | 187(37.8, 33.6-42.2) | 163(33.1, 29.1-37.5) |
|  |  |  | OR = 0.405; p=0.001 | OR = 0.782; p=0.059 | OR = 7.636; p=0.001 |
|  | **2** | **No Trade-off** | 251(53.2, 48.7-57.7) | 181(38.4, 34.1-42.9) | 39(8.2, 6.1-11.1) |
|  |  | **cost trade-off** | 289(61.3, 56.8-65.6) | 146(30.9, 26.9-35.3) | 36(7.6, 5.5-10.4) |
|  |  |  | OR = 1.391; p=0.012 | OR = 0.719; p=0.016 | OR = 0.916; p=0.718 |
| Daily care Needs | **1** | **No Trade-off** | 274(55.6, 51.1-59.9) | 139(28.3, 24.4-32.4) | 79(16.08, 13.08-19.6) |
|  |  | **Effectiveness trade-off** | 172(35.03, 30.9-39.3) | 136(27.6, 23.9-31.8) | 184(37.2, 33.08-41.6) |
|  |  |  | OR = 0.430; p=0.001 | OR = 0.97; p=0.831 | OR = 3.098; p=0.001 |
|  | **2** | **No Trade-off** | 254(53.9, 49.3-58.4) | 154(32.6, 28.5-37.08) | 63(13.3, 10.5-16.7) |
|  |  | **cost trade-off** | 233(49.4, 44.9-53.9) | 157(33.3, 29.2-37.7) | 81(17.1, 14.04-20.8) |
|  |  |  | OR = 0.836; p=0.1709 | OR = 1.029; p=0.835 | OR = 1.345; p=0.1032 |
| Access to Alternative Interventions | **1** | **No Trade-off** | 289(58.6, 54.2-62.9) | 144(29.3, 25.4-33.5) | 59(12.01, 9.4-15.2) |
|  |  | **Effectiveness trade-off** | 208(42.1, 37.8-46.5) | 141(28.7, 24.8-32.8) | 143(29.1, 25.2-33.3) |
|  |  |  | OR = 0.513; p=0.001 | OR = 0.97; p=0.832 | OR = 3.008; p=0.001 |
|  | **2** | **No Trade-off** | 263(55.8, 51.3-60.2) | 141(29.9, 25.9-34.2) | 67(14.2, 11.3-17.6) |
|  |  | **cost trade-off** | 328(69.6, 65.3-73.6) | 95(20.1, 16.7-24.05) | 48(10.1, 7.7-13.2) |
|  |  |  | OR = 1.814; p=0.001 | OR = 0.591; p=0.0005 | OR = 3.684; p=0.058 |
| Individual's Economic status | **1** | **No Trade-off** | 384(78, 74.1-81.4) | 85(17.3, 14.2-20.9) | 23(4.6, 3.1-6.9) |
|  |  | **Effectiveness trade-off** | 283(57.4, 52.9-61.7) | 122(24.8, 21.2-28.8) | 87(17.7, 14.5-21.3) |
|  |  |  | OR = 0.380; p=0.001 | OR = 1.579; p=0.003 | OR = 4.381; p=0.001 |
|  | **2** | **No Trade-off** | 358(76, 71.9-79.6) | 80(16.9, 13.8-20.6) | 33(7, 5.01-9.7) |
|  |  | **cost trade-off** | 367(77.9, 73.9-81.4) | 75(15.9, 12.8-19.5) | 29(6.1, 4.3-8.7) |
|  |  |  | OR = 1.113; p=0.486 | OR = 0.925; p=0.66 | OR = 0.870; p=0.599 |
| Population Size (Common or Rare Diseases) | **1** | **No Trade-off** | 185(37.4, 33.2-41.8) | 152(30.9, 27.01-35.2) | 155(31.5, 27.5-35.8) |
|  |  | **Effectiveness trade-off** | 154(31.3, 27.3-35.6) | 127(25.8, 22.1-29.9) | 211(42.7, 38.4-47.2) |
|  |  |  | OR = 0.762; p=0.0439 | OR = 0.778; p=0.076 | OR = 1.62; p=0.0003 |
|  | **2** | **No Trade-off** | 225(47.7, 43.2-52.3) | 129(27.3, 23.5-31.6) | 117(24.8, 21.1-28.9) |
|  |  | **cost trade-off** | 251(53.2, 48.7-57.7) | 107(22.7, 19.1-26.7) | 113(23.9, 20.3-28.07) |
|  |  |  | OR = 1.247; p=0.0902 | OR = 0.779; p=0.098 | OR = 0.955; p=0.761 |
| Diseases with Absence from work | **1** | **No Trade-off** | 326(66.1, 61.8-70.2) | 116(23.6, 20.06-27.6) | 50(10.1, 7.7-13.1) |
|  |  | **Effectiveness trade-off** | 204(41.3, 37.05-45.7) | 133(27.08, 23.3-31.2) | 155(31.5, 27.5-35.8) |
|  |  |  | OR = 0.360; p=0.001 | OR = 1.201; p=0.212 | OR = 4.068; p=0.001 |
|  | **2** | **No Trade-off** | 344(73.03, 68.8-76.8) | 92(19.5, 16.1-23.3) | 35(7.4, 5.3-10.1) |
|  |  | **cost trade-off** | 336(71.3, 67.07-75.2) | 101(21.4, 17.9-25.3) | 34(7.2, 5.1-9.9) |
|  |  |  | OR = 0.918; p=0.560 | OR = 1.124; p=0.467 | OR = 0.969; p=0.9005 |
| Lifestyle-related diseases | **1** | **No Trade-off** | 208(42.1, 37.8-46.5) | 202(41.1, 36.8-45.5) | 82(16.7, 13.6-20.2) |
|  |  | **Effectiveness trade-off** | 151(30.7, 26.8-34.9) | 160(32.5, 28.5-36.8) | 181(36.6, 32.4-41.03) |
|  |  |  | OR = 0.609; p=0.0002 | OR = 0.691; p=0.005 | OR = 2.886; p=0.001 |
|  | **2** | **No Trade-off** | 224(47.5, 43.06-52.09) | 187(39.7, 35.3-44.2) | 60(12.7, 10.01-16.07) |
|  |  | **cost trade-off** | 249(52.8, 48.3-57.3) | 159(33.7, 29.6-38.1) | 63(13.3, 10.5-16.7) |
|  |  |  | OR = 1.236; p=0.103 | OR = 0.774; p=0.058 | OR = 1.057; p=0.771 |
