# Supplementary material for: Public preferences regarding the priority setting criteria of health interventions for budget allocation: results of a survey of Iranian adults
Source: BMC Public Health. 2022 Nov 8;22:2038. doi: 10.1186/s12889-022-14404-1 (PMC9640781; doi:10.1186/s12889-022-14404-1)
Supplement: Supplementary file 2 — Additional file 2: Supplementary Materials. Questionnaire for Cohort 1. Supplementary Materials. Questionnaire for Cohort 2. [file 12889_2022_14404_MOESM2_ESM.zip › Supp (CBE cohort 2 Quest).docx]

**Supplementary Materials:**

Questionnaire for Cohort 2

**In the name of God**

**In all countries, government and health insurances resources are limited, so they can not financially cover all drugs and treatments. That's why they prioritize drugs and treatments and cover the more important ones. Different drugs and treatments have different properties, for example, some drugs are more effective than others but more expensive. Some medicines are used for incurable patients such as cancer and some for simple diseases such as colds, some medicines are for the elderly and some are for children and etc. Accordingly, we need to set criteria for prioritizing them.**

**The present questionnaire is designed to conduct a study aimed at determining the importance of appropriate criteria for prioritizing drugs and treatment methods. In this study, we aim to know what criteria should be used to prioritize drugs and treatments for financial coverage by the government or insurance funds?. This poll is designed to find out what you think is "fair". The results of this study can be very helpful in "fairer" and "more efficient" the process of allocating funds to drugs and treatment interventions.**

**The stages of conducting and monitoring this study are organized by the Tehran University of Medical Sciences.**

**You have been randomly selected among the Tehran citizens and are eligible to participate in the study. It should be noted that the total number of participants in this study is about 1000 people. Approximate time for answering the questionnaire questions: 20 to 30 minutes**

**Questions:**

**The questionnaire consist of two main parts. In the first part, you will be presented with a number of hypothetical scenarios and you will be asked to determine how the hypothetical budget will be allocated. All scenarios are such that it is assumed that there are a specific number of patients who are quite similar but are divided into two groups based on a specific feature.**

**For example, 200 cancer patients who receive the same treatment have the same effectiveness and cost of treatment, but 100 are adults and 100 are children. You also have a limited budget with which you can treat only 100 patients. Now you have to decide whether to allocate all the budget to one group (for example, only 100 elderly patients) or to divide it in different proportions (for example, 50:50) between the two groups.**

**In the second part, questions related to your demographic status (age, gender, etc.) are asked.**

**Scenario 1-1: Severity of disease**

**Suppose there are two groups of patients who are the same in all aspects, including the cost and effectiveness of treatment, but differ in "disease severity":**

**The first group (100 people) has a "severe" and incurable disease (such as cancer)**

**The second group (100 people) has a "mild" disease.**

**Also, assume that we have a limited budget with which we can treat only 100 patients.**

**With this explanation, how do you want the limited budget that exists to be allocated between the two groups of patients?**

**(Please select from the columns 1 to 7 in the table below, the column that you think is the best possible allocation)**

| **1** | **2** | **3** | **4** | **5** | **6** | **7** |
| --- | --- | --- | --- | --- | --- | --- |
| **All money should be spent on patients with severe disease.** |  |  | **Money should be divided equally between the two groups** |  |  | **All money should be spent on patients with mild disease.** |
| Treatment of 100 patients with severe disease and treatment of 0 patients with mild disease | Treatment of 80 patients with severe disease and treatment of 20 patients with mild disease | Treatment of 60 patients with severe disease and treatment of 40 patients with mild disease | Treatment of 50 patients with severe disease and treatment of 50 patients with mild disease | Treatment of 40 patients with severe disease and treatment of 60 patients with mild disease | Treatment of 20 patients with severe disease and treatment of 80 patients with mild disease | Treatment of 0 patients with severe disease and treatment of 100 patients with mild disease |
| **** | **** | **** | **** | **** | **** | **** |

**Scenario 1-2: Severity of disease (The cost of treatments varies but other features, including effectiveness, are equal)**

**In the previous question, it was assumed that the cost and effectiveness of treatment are the same in the two groups of patients, now suppose there are two drugs (drug A and drug B).**

**Both drugs are similarly safe and effective, but they differ in cost, and the cost of drug A is "twice that" of drug B.**

**Based on the limited budget, we are able to "finally":**

**• Treat 50 patients with "severe" disease using the "more expensive" drug A**

**Or**

**• Treat 100 patients with "mild" disease with "cheaper" drug B**

**Also, assume that we have a limited budget with which we can treat only 100 patients.**

**With this explanation, how do you want the limited budget that exists to be allocated between the two groups of patients?**

**(Please select from the columns 1 to 7 in the table below, the column that you think is the best possible allocation)**

| **1** | **2** | **3** | **4** | **5** | **6** | **7** |
| --- | --- | --- | --- | --- | --- | --- |
| **All money should be spent on drug A: a drug that is used to treat patients with "severe" disease using the "more expensive" drug A** |  |  | **Money should be divided equally between the two groups** |  |  | **All money should be spent on drug B: a drug that is used to treat patients with "mild" disease with "cheaper" drug B** |
| Treatment of 50 patients with severe disease and treatment of 0 patients with mild disease | Treatment of 40 patients with severe disease and treatment of 20 patients with mild disease | Treatment of 30 patients with severe disease and treatment of 40 patients with mild disease | Treatment of 25 patients with severe disease and treatment of 50 patients with mild disease | Treatment of 20 patients with severe disease and treatment of 60 patients with mild disease | Treatment of 10 patients with severe disease and treatment of 80 patients with mild disease | Treatment of 0 patients with severe disease and treatment of 100 patients with mild disease |
| **** | **** | **** | **** | **** | **** | **** |

**Scenario 2-1: Age**

**Suppose there are two groups of patients who are the same in all aspects, including the cost and effectiveness of treatment, But they are different in terms of "age":**

**The first group (100 people) includes "children" patients.**

**The second group (100 people) includes "adult" patients.**

**Also, assume that we have a limited budget with which we can treat only 100 patients.**

**With this explanation, how do you want the limited budget that exists to be allocated between the two groups of patients?**

**(Please select from the columns 1 to 7 in the table below, the column that you think is the best possible allocation)**

| **1** | **2** | **3** | **4** | **5** | **6** | **7** |
| --- | --- | --- | --- | --- | --- | --- |
| **All money should be spent on childeren** |  |  | **Money should be divided equally between the two groups** |  |  | **All money should be spent on adults** |
| Treatment of 100 children and treatment of 0 adult patients | Treatment of 80 children and treatment of 20 adult patients | Treatment of 60 children and treatment of 40 adult patients | Treatment of 50 children and treatment of 50 adult patients | Treatment of 40 children and treatment of 60 adult patients | Treatment of 20 children and treatment of 80 adult patients | Treatment of 0 children and treatment of 100 adult patients |
| **** | **** | **** | **** | **** | **** | **** |

**Scenario 2-2: Age (The cost of treatments varies but other features, including effectiveness, are equal)**

**In the previous question, it was assumed that the cost and effectiveness of treatment are the same in the two groups of patients, now suppose there are two drugs (drug A and drug B).**

**Both drugs are similarly safe and effective, but they differ in cost, and the cost of drug A is "twice that" of drug B.**

**Based on the limited budget, we are able to "finally":**

**• Treat 50 "children" with "more expensive" drug A**

**Or**

**• Treat 100 "adult" patients with "cheaper" drug B**

**Also, assume that we have a limited budget with which we can treat only 100 patients.**

**With this explanation, how do you want the limited budget that exists to be allocated between the two groups of patients?**

**(Please select from the columns 1 to 7 in the table below, the column that you think is the best possible allocation)**

| **1** | **2** | **3** | **4** | **5** | **6** | **7** |
| --- | --- | --- | --- | --- | --- | --- |
| **All money should be spent on drug A: a drug that is used to treat "children" with "more expensive" drug A** |  |  | **Money should be divided equally between the two groups** |  |  | **All money should be spent on drug B: a drug that is used to treat "adult" patients with "cheaper" drug B** |
| Treatment of 50 children and treatment of 0 adult patients | Treatment of 40 children and treatment of 20 adult patients | Treatment of 30 children and treatment of 40 adult patients | Treatment of 25 children and treatment of 50 adult patients | Treatment of 20 children and treatment of 60 adult patients | Treatment of 10 children and treatment of 80 adult patients | Treatment of 0 children and treatment of 100 adult patients |
| **** | **** | **** | **** | **** | **** | **** |

**Scenario 3-1: Daily care needs**

**Suppose there are two groups of patients who are the same in all aspects, including the cost and effectiveness of treatment, But they are different in terms of "need for daily care":**

**In the first group (100 people), patients "depend on a nurse (or family member)" to perform their daily personal tasks during the illness.**

**In the second group (100 people), patients "do not depend on the care of another person" to perform their daily tasks, despite the unpleasant feeling caused by the disease.**

**Also, assume that we have a limited budget with which we can treat only 100 patients.**

**With this explanation, how do you want the limited budget that exists to be allocated between the two groups of patients?**

**(Please select from the columns 1 to 7 in the table below, the column that you think is the best possible allocation)**

| **1** | **2** | **3** | **4** | **5** | **6** | **7** |
| --- | --- | --- | --- | --- | --- | --- |
| **All money should be spent on patients "depend on a nurse (or family member)" to perform their daily personal tasks during the illness** |  |  | **Money should be divided equally between the two groups** |  |  | **All money should be spent on patients "do not depend on the care of another person" to perform their daily tasks** |
| Treatment of 100 patients who are dependent on personal work and treatment of 0 patients who are not dependent on personal work | Treatment of 80 patients who are dependent on personal work and treatment of 20 patients who are not dependent on personal work | Treatment of 60 patients who are dependent on personal work and treatment of 40 patients who are not dependent on personal work | Treatment of 50 patients who are dependent on personal work and treatment of 50 patients who are not dependent on personal work | Treatment of 40 patients who are dependent on personal work and treatment of 60 patients who are not dependent on personal work | Treatment of 20 patients who are dependent on personal work and treatment of 80 patients who are not dependent on personal work | Treatment of 0 patients who are dependent on personal work and treatment of 100 patients who are not dependent on personal work |
| **** | **** | **** | **** | **** | **** | **** |

**Scenario 3-2: Daily care needs (The cost of treatments varies but other features, including effectiveness, are equal)**

**In the previous question, it was assumed that the cost and effectiveness of treatment are the same in the two groups of patients, now suppose there are two drugs (drug A and drug B).**

**Both drugs are similarly safe and effective, but they differ in cost, and the cost of drug A is "twice that" of drug B.**

**Based on the limited budget, we are able to "finally":**

**• Treat 50 patients who are "dependent" on other people for daily tasks due to illness with "more expensive" drug A.**

**Or**

**• Treat 100 patients who are "not dependent" on other people for daily tasks due to illness with "cheaper" drug B.**

**Also, assume that we have a limited budget with which we can treat only 100 patients.**

**With this explanation, how do you want the limited budget that exists to be allocated between the two groups of patients?**

**(Please select from the columns 1 to 7 in the table below, the column that you think is the best possible allocation)**

| **1** | **2** | **3** | **4** | **5** | **6** | **7** |
| --- | --- | --- | --- | --- | --- | --- |
| **All money should be spent on drug A: a drug that is used in patients who are "dependent" on other people for daily tasks due to illness with "more expensive" drug A** |  |  | **Money should be divided equally between the two groups** |  |  | **All money should be spent on drug B: a drug that is used in patients who are "not dependent" on other people for daily tasks due to illness with "cheaper" drug B** |
| Treatment of 50 patients who are dependent on personal work and treatment of 0 patients who are not dependent on personal work | Treatment of 40 patients who are dependent on personal work and treatment of 20 patients who are not dependent on personal work | Treatment of 30 patients who are dependent on personal work and treatment of 40 patients who are not dependent on personal work | Treatment of 25 patients who are dependent on personal work and treatment of 50 patients who are not dependent on personal work | Treatment of 20 patients who are dependent on personal work and treatment of 60 patients who are not dependent on personal work | Treatment of 10 patients who are dependent on personal work and treatment of 80 patients who are not dependent on personal work | Treatment of 0 patients who are dependent on personal work and treatment of 100 patients who are not dependent on personal work |
| **** | **** | **** | **** | **** | **** | **** |

**Scenario 4-1: Access to alternative interventions**

**Suppose there are two groups of patients who are the same in all aspects, including the cost and effectiveness of treatment, But they are different in terms of "** **access to alternative interventions ":**

**• For the first group (100 people) there is only "one treatment (drug)".**

**• For the second group (100 people) there are "several treatments (drugs)".**

**Also, assume that we have a limited budget with which we can treat only 100 patients.**

**With this explanation, how do you want the limited budget that exists to be allocated between the two groups of patients?**

**(Please select from the columns 1 to 7 in the table below, the column that you think is the best possible allocation)**

| **1** | **2** | **3** | **4** | **5** | **6** | **7** |
| --- | --- | --- | --- | --- | --- | --- |
| **All money should be spent on patients for whom there is only one treatment** |  |  | **Money should be divided equally between the two groups** |  |  | **All the money should be spent on patients for whom there are several treatments** |
| Treatment of 100 patients for whom there is only one treatment and treatment of 0 patients for whom there are several treatments | Treatment of 80 patients for whom there is only one treatment and treatment of 20 patients for whom there are several treatments | Treatment of 60 patients for whom there is only one treatment and treatment of 40 patients for whom there are several treatments | Treatment of 50 patients for whom there is only one treatment and treatment of 50 patients for whom there are several treatments | Treatment of 40 patients for whom there is only one treatment and treatment of 60 patients for whom there are several treatments | Treatment of 20 patients for whom there is only one treatment and treatment of 80 patients for whom there are several treatments | Treatment of 0 patients for whom there is only one treatment and treatment of 100 patients for whom there are several treatments |
| **** | **** | **** | **** | **** | **** | **** |

**Scenario 4-2: Access to alternative interventions (The cost of treatments varies but other features, including effectiveness, are equal)**

**In the previous question, it was assumed that the cost and effectiveness of treatment are the same in the two groups of patients, now suppose there are two drugs (drug A and drug B).**

**Both drugs are similarly safe and effective, but they differ in cost, and the cost of drug A is "twice that" of drug B.**

**Based on the limited budget, we are able to "finally":**

**• Treat 50 patients for whom "there is only one treatment (drug)" with "more expensive" drug A.**

**Or**

**• Treat 100 patients for whom there are "several treatments" with "cheaper" drug B.**

**Also, assume that we have a limited budget with which we can treat only 100 patients.**

**With this explanation, how do you want the limited budget that exists to be allocated between the two groups of patients?**

**(Please select from the columns 1 to 7 in the table below, the column that you think is the best possible allocation)**

| **1** | **2** | **3** | **4** | **5** | **6** | **7** |
| --- | --- | --- | --- | --- | --- | --- |
| **All money should be spent on drug A: a drug that is used in patients for whom "there is only one treatment (drug)" with "more expensive" drug A** |  |  | **Money should be divided equally between the two groups** |  |  | **All money should be spent on drug B: a drug that is used in patients for whom there are "several treatments" with "cheaper" drug B** |
| Treatment of 50 patients for whom there is only one treatment and treatment of 0 patients for whom there are several treatments | Treatment of 40 patients for whom there is only one treatment and treatment of 20 patients for whom there are several treatments | Treatment of 30 patients for whom there is only one treatment and treatment of 40 patients for whom there are several treatments | Treatment of 25 patients for whom there is only one treatment and treatment of 50 patients for whom there are several treatments | Treatment of 20 patients for whom there is only one treatment and treatment of 60 patients for whom there are several treatments | Treatment of 10 patients for whom there is only one treatment and treatment of 80 patients for whom there are several treatments | Treatment of 0 patients for whom there is only one treatment and treatment of 100 patients for whom there are several treatments |
| **** | **** | **** | **** | **** | **** | **** |

**Scenario 5-1: Economic status of patients**

**Suppose there are two groups of patients who are the same in all aspects, including the cost and effectiveness of treatment, But they are different in terms of "** **economic status of patients ":**

**The first group (100 people) includes patients who are "economically disadvantaged". (People from the "lower" income deciles)**

**The second group (100 people) includes patients who are "economically well off". (People from the "upper" income deciles)**

**Also, assume that we have a limited budget with which we can treat only 100 patients.**

**With this explanation, how do you want the limited budget that exists to be allocated between the two groups of patients?**

**(Please select from the columns 1 to 7 in the table below, the column that you think is the best possible allocation)**

| **1** | **2** | **3** | **4** | **5** | **6** | **7** |
| --- | --- | --- | --- | --- | --- | --- |
| **All money should be spent on patients who are "economically disadvantaged"** |  |  | **Money should be divided equally between the two groups** |  |  | **All the money should be spent on patients who are " economically well off"** |
| Treatment of 100 economically disadvantaged patients and treatment of 0 economically well off patients | Treatment of 80 economically disadvantaged patients and treatment of 20 economically well off patients | Treatment of 60 economically disadvantaged patients and treatment of 40 economically well off patients | Treatment of 50 economically disadvantaged patients and treatment of 50 economically well off patients | Treatment of 40 economically disadvantaged patients and treatment of 60 economically well off patients | Treatment of 20 economically disadvantaged patients and treatment of 80 economically well off patients | Treatment of 0 economically disadvantaged patients and treatment of 100 economically well off patients |
| **** | **** | **** | **** | **** | **** | **** |

**Scenario 5-2: Economic status of patients (The cost of treatments varies but other features, including effectiveness, are equal)**

**In the previous question, it was assumed that the cost and effectiveness of treatment are the same in the two groups of patients, now suppose there are two drugs (drug A and drug B).**

**Both drugs are similarly safe and effective, but they differ in cost, and the cost of drug A is "twice that" of drug B.**

**Based on the limited budget, we are able to "finally":**

**• Treat 50 patients who are "economically disadvantaged" with "more expensive" drug A.**

**Or**

**• Treat 100 patients who are "** **economically well off " with "cheaper" drug B.**

**Also, assume that we have a limited budget with which we can treat only 100 patients.**

**With this explanation, how do you want the limited budget that exists to be allocated between the two groups of patients?**

**(Please select from the columns 1 to 7 in the table below, the column that you think is the best possible allocation)**

| **1** | **2** | **3** | **4** | **5** | **6** | **7** |
| --- | --- | --- | --- | --- | --- | --- |
| **All money should be spent on drug A: a drug that is used in patients who are "economically disadvantaged" with "more expensive" drug A** |  |  | **Money should be divided equally between the two groups** |  |  | **All money should be spent on drug B: a drug that is used in patients who are " economically well off " with "cheaper" drug B** |
| Treatment of 50 economically disadvantaged patients and treatment of 0 economically well off patients | Treatment of 40 economically disadvantaged patients and treatment of 20 economically well off patients | Treatment of 30 economically disadvantaged patients and treatment of 40 economically well off patients | Treatment of 25 economically disadvantaged patients and treatment of 50 economically well off patients | Treatment of 20 economically disadvantaged patients and treatment of 60 economically well off patients | Treatment of 10 economically disadvantaged patients and treatment of 80 economically well off patients | Treatment of 0 economically disadvantaged patients and treatment of 100 economically well off patients |
| **** | **** | **** | **** | **** | **** | **** |

**Scenario 6-1: Common or rare diseases**

**Suppose there are two groups of patients who are the same in all aspects, including the cost and effectiveness of treatment, But they differ in "patient population size (common or rare diseases)":**

**The first group (100 people) includes patients with one of the "rare diseases" (diseases that usually affect less than 2,000 people in Iran).**

**The second group (100 people) includes patients with one of the "common diseases" (diseases that usually affect more than 500,000 people in Iran).**

**Also, assume that we have a limited budget with which we can treat only 100 patients.**

**With this explanation, how do you want the limited budget that exists to be allocated between the two groups of patients?**

**(Please select from the columns 1 to 7 in the table below, the column that you think is the best possible allocation)**

| **1** | **2** | **3** | **4** | **5** | **6** | **7** |
| --- | --- | --- | --- | --- | --- | --- |
| **All money should be spent on treating patients with rare diseases** |  |  | **Money should be divided equally between the two groups** |  |  | **All money should be spent on treating patients with common diseases** |
| Treatment of 100 patients with rare diseases and treatment of 0 patients with common diseases | Treatment of 80 patients with rare diseases and treatment of 20 patients with common diseases | Treatment of 60 patients with rare diseases and treatment of 40 patients with common diseases | Treatment of 50 patients with rare diseases and treatment of 50 patients with common diseases | Treatment of 40 patients with rare diseases and treatment of 60 patients with common diseases | Treatment of 20 patients with rare diseases and treatment of 80 patients with common diseases | Treatment of 0 patients with rare diseases and treatment of 100 patients with common diseases |
| **** | **** | **** | **** | **** | **** | **** |

**Scenario 6-2: Common or rare diseases (The cost of treatments varies but other features, including effectiveness, are equal)**

**In the previous question, it was assumed that the cost and effectiveness of treatment are the same in the two groups of patients, now suppose there are two drugs (drug A and drug B).**

**Both drugs are similarly safe and effective, but they differ in cost, and the cost of drug A is "twice that" of drug B.**

**Based on the limited budget, we are able to "finally":**

**• Treat 50 patients with "rare diseases" with "more expensive" drug A.**

**Or**

**• Treat 100 patients with "common diseases" with "cheaper" drug B.**

**Also, assume that we have a limited budget with which we can treat only 100 patients.**

**With this explanation, how do you want the limited budget that exists to be allocated between the two groups of patients?**

**(Please select from the columns 1 to 7 in the table below, the column that you think is the best possible allocation)**

| **1** | **2** | **3** | **4** | **5** | **6** | **7** |
| --- | --- | --- | --- | --- | --- | --- |
| **All money should be spent on drug A: a drug that is used in patients with "rare diseases" with "more expensive" drug A** |  |  | **Money should be divided equally between the two groups** |  |  | **All money should be spent on drug B: a drug that is used in patients with "common diseases" with "cheaper" drug B** |
| Treatment of 50 patients with rare diseases and treatment of 0 patients with common diseases | Treatment of 40 patients with rare diseases and treatment of 20 patients with common diseases | Treatment of 30 patients with rare diseases and treatment of 40 patients with common diseases | Treatment of 25 patients with rare diseases and treatment of 50 patients with common diseases | Treatment of 20 patients with rare diseases and treatment of 60 patients with common diseases | Treatment of 10 patients with rare diseases and treatment of 80 patients with common diseases | Treatment of 0 patients with rare diseases and treatment of 100 patients with common diseases |
| **** | **** | **** | **** | **** | **** | **** |

**Scenario 7-1: Absence from work**

**Suppose there are two groups of patients who are the same in all aspects, including the cost and effectiveness of treatment, But they are different in terms of "work absenteeism rate":**

**The first group (100 people) includes patients whose disease are among the diseases that "affect the patient's work" (diseases that lead to absenteeism).**

**The second group (100 people) includes patients whose disease are among the diseases that "do not affect the patient's work" (diseases that do not lead to absenteeism).**

**Also, assume that we have a limited budget with which we can treat only 100 patients.**

**With this explanation, how do you want the limited budget that exists to be allocated between the two groups of patients?**

**(Please select from the columns 1 to 7 in the table below, the column that you think is the best possible allocation)**

| **1** | **2** | **3** | **4** | **5** | **6** | **7** |
| --- | --- | --- | --- | --- | --- | --- |
| **All the money should be spent on treating patients with diseases that lead to work absenteeism** |  |  | **Money should be divided equally between the two groups** |  |  | **All the money should be spent on treating patients with diseases that do not lead to work absenteeism** |
| Treatment of 100 patients with diseases that lead to work absenteeism and treatment of 0 patients with diseases that do not lead to work absenteeism | Treatment of 80 patients with diseases that lead to work absenteeism and treatment of 20 patients with diseases that do not lead to work absenteeism | Treatment of 60 patients with diseases that lead to work absenteeism and treatment of 40 patients with diseases that do not lead to work absenteeism | Treatment of 50 patients with diseases that lead to work absenteeism and treatment of 50 patients with diseases that do not lead to work absenteeism | Treatment of 40 patients with diseases that lead to work absenteeism and treatment of 60 patients with diseases that do not lead to work absenteeism | Treatment of 20 patients with diseases that lead to work absenteeism and treatment of 80 patients with diseases that do not lead to work absenteeism | Treatment of 0 patients with diseases that lead to work absenteeism and treatment of 100 patients with diseases that do not lead to work absenteeism |
| **** | **** | **** | **** | **** | **** | **** |

**Scenario 7-2: Absence from work (The cost of treatments varies but other features, including effectiveness, are equal)**

**In the previous question, it was assumed that the cost and effectiveness of treatment are the same in the two groups of patients, now suppose there are two drugs (drug A and drug B).**

**Both drugs are similarly safe and effective, but they differ in cost, and the cost of drug A is "twice that" of drug B.**

**Based on the limited budget, we are able to "finally":**

**• Treat 50 patients whose disease "affects the patient's work" with the "more expensive" drug A.**

**Or**

**• Treat 100 patients whose disease "does not affect the patient's work" with the "cheaper" drug B.**

**Also, assume that we have a limited budget with which we can treat only 100 patients.**

**With this explanation, how do you want the limited budget that exists to be allocated between the two groups of patients?**

**(Please select from the columns 1 to 7 in the table below, the column that you think is the best possible allocation)**

| **1** | **2** | **3** | **4** | **5** | **6** | **7** |
| --- | --- | --- | --- | --- | --- | --- |
| **All money should be spent on drug A: a drug that is used in patients whose disease "affects the patient's work" with the "more expensive" drug A** |  |  | **Money should be divided equally between the two groups** |  |  | **All money should be spent on drug B: a drug that is used in patients whose disease "does not affect the patient's work" with the "cheaper" drug B** |
| Treatment of 50 patients with diseases that lead to work absenteeism and treatment of 0 patients with diseases that do not lead to work absenteeism | Treatment of 40 patients with diseases that lead to work absenteeism and treatment of 20 patients with diseases that do not lead to work absenteeism | Treatment of 30 patients with diseases that lead to work absenteeism and treatment of 40 patients with diseases that do not lead to work absenteeism | Treatment of 25 patients with diseases that lead to work absenteeism and treatment of 50 patients with diseases that do not lead to work absenteeism | Treatment of 20 patients with diseases that lead to work absenteeism and treatment of 60 patients with diseases that do not lead to work absenteeism | Treatment of 10 patients with diseases that lead to work absenteeism and treatment of 80 patients with diseases that do not lead to work absenteeism | Treatment of 0 patients with diseases that lead to work absenteeism and treatment of 100 patients with diseases that do not lead to work absenteeism |
| **** | **** | **** | **** | **** | **** | **** |

**Scenario 8-1: Lifestyle related diseases**

**Suppose there are two groups of patients who are the same in all aspects, including the cost and effectiveness of treatment, But they differ in "the relationship between the disease and the indviduals lifestyle ":**

**The first group (100 people) includes patients whose disease is one of the diseases that "have no relation to the unhealthy lifestyle of people" (such as genetic diseases).**

**The second group (100 people) includes patients whose diseases are among the diseases that "have a clear association with an unhealthy lifestyle" (such as type 2 diabetes, many heart diseases).**

**Also, assume that we have a limited budget with which we can treat only 100 patients.**

**With this explanation, how do you want the limited budget that exists to be allocated between the two groups of patients?**

**(Please select from the columns 1 to 7 in the table below, the column that you think is the best possible allocation)**

| **1** | **2** | **3** | **4** | **5** | **6** | **7** |
| --- | --- | --- | --- | --- | --- | --- |
| **All money should be spent on treating patients with diseases unrelated to unhealthy lifestyles** |  |  | **Money should be divided equally between the two groups** |  |  | **All the money should be spent on treating patients with diseases related to unhealthy lifestyle** |
| Treatment of 100 patients with unrelated lifestyle diseases and treatment of 0 patients with diseases related to unhealthy lifestyle | Treatment of 100 patients with unrelated lifestyle diseases and treatment of 0 patients with diseases related to unhealthy lifestyle | Treatment of 100 patients with unrelated lifestyle diseases and treatment of 0 patients with diseases related to unhealthy lifestyle | Treatment of 100 patients with unrelated lifestyle diseases and treatment of 0 patients with diseases related to unhealthy lifestyle | Treatment of 100 patients with unrelated lifestyle diseases and treatment of 0 patients with diseases related to unhealthy lifestyle | Treatment of 100 patients with unrelated lifestyle diseases and treatment of 0 patients with diseases related to unhealthy lifestyle | Treatment of 100 patients with unrelated lifestyle diseases and treatment of 0 patients with diseases related to unhealthy lifestyle |
| **** | **** | **** | **** | **** | **** | **** |

**Scenario 8-2: Lifestyle related diseases (The cost of treatments varies but other features, including effectiveness, are equal)**

**In the previous question, it was assumed that the cost and effectiveness of treatment are the same in the two groups of patients, now suppose there are two drugs (drug A and drug B).**

**Both drugs are similarly safe and effective, but they differ in cost, and the cost of drug A is "twice that" of drug B.**

**Based on the limited budget, we are able to "finally":**

**• Treat 50 patients with a disease that "not related unhealthy lifestyle" using the "more expensive" drug A.**

**Or**

**• Treat 100 patients with a disease whose "related unhealthy lifestyle" using the "cheaper" drug B.**

**Also, assume that we have a limited budget with which we can treat only 100 patients.**

**With this explanation, how do you want the limited budget that exists to be allocated between the two groups of patients?**

**(Please select from the columns 1 to 7 in the table below, the column that you think is the best possible allocation)**

| **1** | **2** | **3** | **4** | **5** | **6** | **7** |
| --- | --- | --- | --- | --- | --- | --- |
| **All money should be spent on drug A: a drug that is used in patients with a disease that "not related unhealthy lifestyle" using the "more expensive" drug A** |  |  | **Money should be divided equally between the two groups** |  |  | **All money should be spent on drug B: a drug that is used in patients with a disease whose "related unhealthy lifestyle" using the "cheaper" drug B** |
| Treatment of 50 patients with unrelated lifestyle diseases and treatment of 0 patients with diseases related to unhealthy lifestyle | Treatment of 40 patients with unrelated lifestyle diseases and treatment of 20 patients with diseases related to unhealthy lifestyle | Treatment of 30 patients with unrelated lifestyle diseases and treatment of 40 patients with diseases related to unhealthy lifestyle | Treatment of 25 patients with unrelated lifestyle diseases and treatment of 50 patients with diseases related to unhealthy lifestyle | Treatment of 20 patients with unrelated lifestyle diseases and treatment of 60 patients with diseases related to unhealthy lifestyle | Treatment of 10 patients with unrelated lifestyle diseases and treatment of 80 patients with diseases related to unhealthy lifestyle | Treatment of 0 patients with unrelated lifestyle diseases and treatment of 100 patients with diseases related to unhealthy lifestyle |
| **** | **** | **** | **** | **** | **** | **** |
